# EPAC2 acts as a negative regulator in Matrigel-driven tubulogenesis of human microvascular endothelial cells

**DOI:** 10.1038/s41598-021-98906-9

**Published:** 2021-09-30

**Authors:** Takayuki Ikeda, Yoshino Yoshitake, Yasuo Yoshitomi, Hidehito Saito-Takatsuji, Yasuhito Ishigaki, Hideto Yonekura

**Affiliations:** 1 Department of Biochemistry, Kanazawa Medical University School of Medicine, 1-1 Daigaku, Uchinada, Kahoku-gun, Ishikawa 920-0293 Japan; 2 Division of Molecular and Cell Biology, Medical Research Institute, Kanazawa Medical University, 1-1 Daigaku, Uchinada, Kahoku-gun, Ishikawa 920-0293 Japan

**Keywords:** Angiogenesis, Cell biology, Molecular biology

## Abstract

Angiogenesis is physiologically essential for embryogenesis and development and reinitiated in adult animals during tissue growth and repair. Forming new vessels from the walls of existing vessels occurs as a multistep process coordinated by sprouting, branching, and a new lumenized network formation. However, little is known regarding the molecular mechanisms that form new tubular structures, especially molecules regulating the proper network density of newly formed capillaries. This study conducted microarray analyses in human primary microvascular endothelial cells (HMVECs) plated on Matrigel. The *RAPGEF4* gene that encodes exchange proteins directly activated by cAMP 2 (EPAC2) proteins was increased in Matrigel-driven tubulogenesis. Tube formation was suppressed by the overexpression of EPAC2 and enhanced by EPAC2 knockdown in endothelial cells. Endothelial cell morphology was changed to round cell morphology by EPAC2 overexpression, while EPAC2 knockdown showed an elongated cell shape with filopodia-like protrusions. Furthermore, increased EPAC2 inhibited endothelial cell migration, and ablation of EPAC2 inversely enhanced cell mobility. These results suggest that EPAC2 affects the morphology and migration of microvascular endothelial cells and is involved in the termination and proper network formation of vascular tubes.

## Introduction

Angiogenesis, the process by which new vascular networks are formed from preexisting capillaries, is physiologically essential for embryogenesis and development. It is reinitiated in adult animals during tissue growth and repair processes, such as wound healing and menstrual cycle^1^. It also plays a vital role in the progression of various pathological conditions, such as cancer growth and metastasis, diabetic retinopathy, and rheumatoid arthritis^1^. The formation of new vessels from the walls of existing vessels occurs as a multistep process coordinated by sprouting, branching, and new lumenized network formation. Vascular endothelial growth factor (VEGF) is the most potent angiogenic activator, and the initiation and progression of vessel formation are now well understood^2^. The tip cells, which produce many filopodia, lead to sprouting; and the stalk cells follow the tip cells. Consequently, the tight endothelial cell–cell adhesion is disrupted, and the stalk cells proliferate and migrate to form the vascular lumen. It has been reported that the tip and stalk cells differ in their gene expression profile, suggesting that they have specialized functions during sprouting angiogenesis^2^. However, little is known regarding the molecular mechanisms in the tubular structure formation, especially molecules in terminating angiogenesis and proper network formation of vascular tubes.

Several in vitro models have been developed to investigate the angiogenic states^3,4^. Specific angiogenesis models of angiogenesis are the capillary-like structure formation of endothelial cells^5^. Endothelial cells can form tubules on a gel composed of extracted basement membrane derived from mouse Engelbreth–Kolm–Swarm sarcoma (Matrigel). The Matrigel, whose primary component is laminin, can initiate endothelial cell tube formation. The capillary-like structure of endothelial cells contains a lumen surrounded by cells. The advantage of this model is that the molecular dissection of the tube formation can be unveiled because the differentiated cells are compared with undifferentiated, proliferating cells. Some studies have been conducted to investigate the differential gene expression in endothelial cells using the Matrigel^6–8^. Although these studies have described some candidate genes as relevant to the tube formation, crucial genes remain unidentified.

Exchange proteins directly activated by cAMP (EPAC) is a family of guanine nucleotide exchange factor (GEF) for the small GTPases, Raps (Rap1 and Rap2)^9^. EPACs activate Raps by stimulating guanine nucleotide exchange in a cAMP-dependent manner. Raps regulate actin cytoskeletal dynamics, such as integrin-mediated cell adhesion and cadherin-mediated formation of cell junctions^10,11^. There are two isoforms of EPAC, EPAC1 and EPAC2, coded by *RAPGEF3* and *RAPGEF4* genes, respectively. Although their amino acid similarity is less than 50%, they have the same regulatory and catalytic domains^9,12,13^. cAMP binding to the regulatory domain induces a dynamic conformational change and stabilizes the open conformation, allowing the catalytic domain to interact with the substrate, Raps. EPAC1 expression is relatively ubiquitous^14^ and involved in a myriad of physiological functions. Notably, functional roles in the cardiovascular system are well-studied^15^. EPAC2 is primarily expressed in the brain and adrenal gland with limited levels in the heart, small intestine, and testis^14^. The prominent roles of EPAC2 are insulin and glucagon secretion in the pancreatic islets and neurotransmitter release in the brain^13,16–18^. However, EPAC2 expressions and functions in endothelial cells remain elusive.

This study investigated the differential gene expression of tube-forming endothelial cells versus monolayer cultured cells using microarray analysis and identified EPAC2 as a novel angiogenic regulator. EPAC2 regulates endothelial cell morphology and cell migration activity and negatively controls the angiogenic processes, resulting in adequate vascular networks.

## Results

### Microarray analysis of gene expression during the tube formation of human primary microvascular endothelial cells (HMVECs)

**Figure 1:**
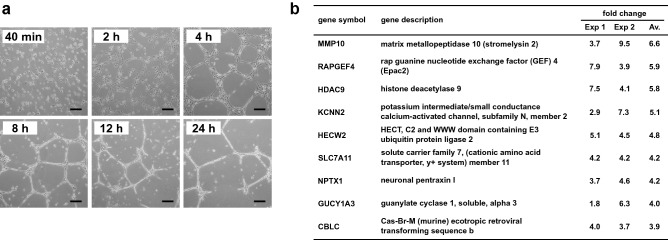
Gene expression analysis of HMVECs during in vitro tube formation on Matrigel. (**a**) HMVECs were plated on Matrigel and observed at the indicated time. Cells were cultured in 0.5% FBS/HMEB2 medium containing 30 ng/mL of VEGF. Scale bars: 200 μm. (**b**) Gene expression profiling of HMVECs in Matrigel-driven tubulogenesis was analyzed by DNA microarray. Tube formation of HMVECs was performed in 0.5% FBS/HMEB2 medium containing 30 ng/mL of VEGF. Total RNAs from 8 h after cell culture were prepared, and DNA microarray analyses were performed as described in the Methods section. Gene expression signals during tube formation were compared with those of monolayer cultured cells. Listed genes showed higher fold changes of the average (Av.) of the two independent experiments (Exp 1 and Exp 2).

HMVECs form capillary-like structures on the Matrigel (Matrigel-driven tubulogenesis). It comprises of an early attachment and migration phase lasting for approximately 2 h and reorganization and capillary formation phase extending for 2–8 h followed by a breakdown after 24 h (Fig. 1a). To identify genes that are involved in capillary-like structure formation in HMVECs, we performed microarray analyses. HMVECs on Matrigel after 8 h post-plating were compared with monolayer cultured HMVECs which were inoculated on a plate. One hundred and fifty-one genes were differentially expressed in the two independent experiments with more than a 1.8-fold change (Supplementary Table S1 online). Among them, 87 genes were upregulated, and 64 genes were downregulated. According to previous reports^19,20^, matrix metallopeptidase 10 (MMP10) expression was relatively highly upregulated (Fig. 1b). Notably, the *RAPGEF4* gene, which encodes the EPAC2 proteins, was increased in Matrigel-driven tubulogenesis (Fig. 1b). EPAC2 has not been identified as a tubulogenesis-related protein so far. Furthermore, Rap1, a substrate of EPAC2, regulates actin cytoskeletal dynamics and is involved in angiogenesis^10^. Thus, we focused on EPAC2 and analyzed its functions in tube formation.

### EPAC2 is upregulated in Matrigel-driven tubulogenesis

**Figure 2:**
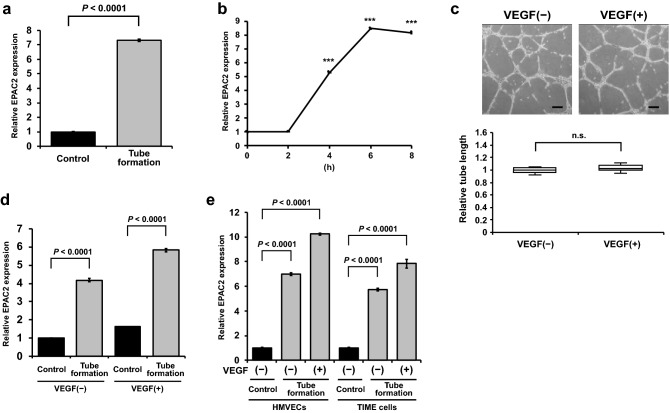
EPAC2 expression in endothelial cells during in vitro tube formation. (**a**) RT-qPCR confirmed EPAC2 expression during tube formation. Data were expressed as mean ± S.E.M. (n = 3). (**b**) Time-course experiment of the EPAC2 expression. HMVECs were cultured on Matrigel in the presence of VEGF, and total RNAs were prepared at the indicated time. Data were expressed as mean ± S.E.M. (n = 3). ***P < 0.001 versus 0 h. (**c**) VEGF is not necessary for the tube formation of HMVECs. HMVECs were cultured on Matrigel in the presence or absence of VEGF, and tube lengths were analyzed at 8 h as described in the Methods section (n = 10). The box represents the 25–75th percentiles, and the median is indicated. The whiskers show the maximum and minimum values. Scale bars: 200 μm. (**d**) EPAC2 expression was increased by tube formation but not VEGF. HMVECs were cultured on Matrigel in the presence or absence of VEGF for 8 h, and RT-qPCR was used to analyze EPAC2 mRNA levels. Data were expressed as mean ± S.E.M. (n = 3). (**e**) EPAC2 expression was increased in HMVECs and TIME cells. HMVECs and TIME cells were cultured on Matrigel in the presence or absence of VEGF for 8 h, and RT-qPCR was used to analyze EPAC2 mRNA levels. Data were expressed as mean ± S.E.M. (n = 3).

RT-qPCR was conducted to confirm the upregulation of EPAC2 during tube formation. EPAC2 expression during tubulogenesis was increased 7.3-fold compared with the control monolayer cultured HMVECs (Fig. 2a). In the time-course experiment, EPAC2 was induced at 2 h after plating on Matrigel and peaked at 6–8 h, suggesting that EPAC2 expression is not induced by Matrigel but driven during capillary formation and reorganization (Fig. 2b). Matrigel-driven tubulogenesis experiments shown in Figs. 1a and 2a were conducted in the presence of VEGF, but the monolayer HMVECs, the control, were cultured without VEGF. Thus, we examined whether VEGF is necessary for forming capillary-like structures in endothelial cells. When HMVECs were cultured on Matrigel with or without VEGF, there was no difference in the tube length (Fig. 2c). Furthermore, EPAC2 was upregulated during tube formation with and without VEGF (Fig. 2d), suggesting that EPAC2 upregulation depends on tube formation itself but not VEGF. We also observed an increase in EPAC2 expression in telomerase-immortalized microvascular endothelial (TIME) cells (Fig. 2e). These data suggest that EPAC2 is involved in tube formation in microvascular endothelial cells.

### EPAC2B and EPAC2C isoforms are induced during tube formation

**Figure 3:**
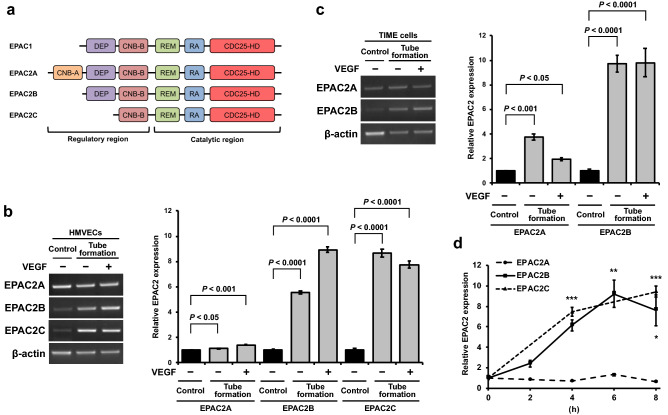
EPAC2 isoform expression in endothelial cells in the in vitro tube formation. (**a**) Schematic representation of EPAC1 and EPAC2 isoforms. EPAC1 and EPAC2 have an N-terminal regulatory region and a C-terminal catalytic region. The regulatory region comprises one or two cyclic nucleotide binding domains (CNB) and a DEP (Disheveled, Egl-10, and Pleckstrin) domain. The catalytic region contains a Ras exchange motif (REM), a Ras association (RA) domain, and a CDC25 homology domain (CDC25-HD). EPAC2A has two CNBs, but EPAC1, EPAC2B, and EPAC2C only have a CNB. (**b,c**) RT-PCR analysis of EPAC2 isoforms using isoform-specific primers. HMVECs (**b**) and TIME cells (**c**) were cultured on Matrigel in the presence or absence of VEGF for 8 h, and RT-PCR was performed using variant-specific primers. Band intensity was quantified, and data were expressed as mean ± S.E.M. (n = 3). (**d**) Time-course experiment of the EPAC2 isoform expression. HMVECs were cultured on Matrigel in the presence of VEGF, and total RNAs were prepared at the indicated time. Band intensity was quantified and data were expressed as mean ± S.E.M. (n = 3). *P < 0.05, **P < 0.01, and ***P < 0.001 versus 0 h. Full-length gels are presented in Supplementary Fig. S4.

There are two EPAC isoforms, EPAC1 and EPAC2 (Fig. 3a). In our experiments, only EPAC2 was upregulated, and EPAC1 was not upregulated during tube formation in HMVECs and TIME cells (Supplementary Fig. S1 online). EPAC2 has three variants, EPAC2A, EPAC2B, and EPAC2C (Fig. 3a). To examine which variant is induced during tube formation, RT-PCR was conducted using specific primers for each variant (Table 1 and Supplementary Fig. S2 online). EPAC2A was slightly increased during tube formation in HMVECs (Fig. 3b) and TIME cells (Fig. 3c). In contrast, EPAC2B and EPAC2C expressions were enhanced during tube formation, suggesting that EPAC2B and EPAC2C are isoforms induced during tube formation (Fig. 3b,c, and Supplementary Fig. S3 online). Furthermore, the induction of these isoforms was independent of VEGF in HMVECs and TIME cells (Fig. 3b,c). The time-course study also demonstrated that EPAC2B and EPAC2C were increased at 2 h after plating on Matrigel and peaked at 6–8 h, as seen in Fig. 2b (Fig. 3d). These results indicated that EPAC2B and EPAC2C are involved in tube formation of microvascular endothelial cells.

**Table 1:** Primers for RT-PCR and oligonucleotides for shRNA.

Target gene	Sequence
RAPGEF4 variant 1 (EPAC2A)	5′-GATCCAGCGAAGATGTGGAT-3′
	5′-CACCATAAGGAGGAGCCAGA-3′
RAPGEF4 variant 2 (EPAC2B)	5′-AACCATCAGCACCAGTTTCC-3′
	5′-CACCATAAGGAGGAGCCAGA-3′
RAPGEF4 variant 3 (EPAC2C)	5′-GCTGACACGTGCTTCCATGT-3′
	5′-GTCATCCACAGTCCTCTGGCCA-3′
shRNA for control	5′-TAGCGACTAAACACATCAA-3′
shRNA for EPAC2	5′-TCAGTGAATGTAGTCATTT-3′
	5′-GTTCATTGACAATCTAGTAAA-3′
	5′-GAGTTATGTACGGCAATTAAA-3′

### EPAC2 suppresses tube formation of microvascular endothelial cells

**Figure 4:**
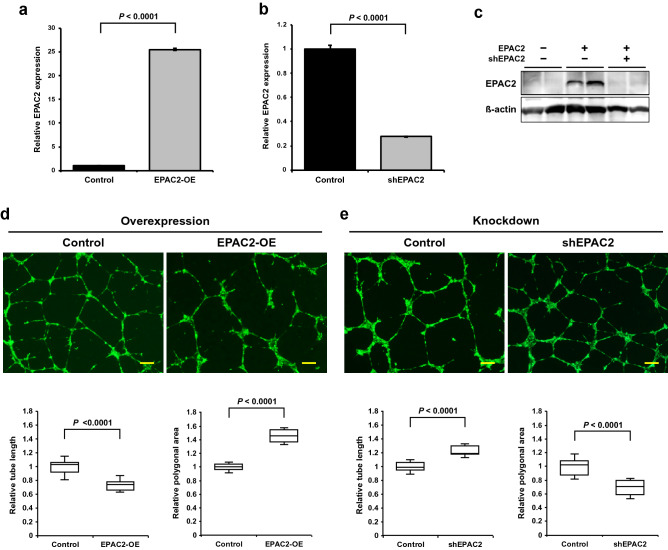
EPAC2 modulates tube formation on Matrigel. (**a,b**) EPAC2 expression in EPAC2 overexpressing (**a**) and knockdown (**b**) TIME cells. Data were expressed as mean ± S.E.M. (n = 3). (**c**) Protein levels of EPAC2 expression. EPAC2 suppression was confirmed in EPAC2 overexpressing HEK293T cells. (**d,e**) Tube formation was suppressed by EPAC2 overexpression (**d**) and facilitated by EPAC2 knockdown (**e**). EPAC2 overexpressing or knockdown TIME cells were cultured on Matrigel in the absence of VEGF, and tube lengths and polygonal areas were analyzed at 8 h as described in the Methods section (n = 10). The box represents the 25–75th percentiles, and the median is indicated. The whiskers show the maximum and minimum values. The same results were obtained in at least three independent experiments. Scale bars: 200 μm. Full-length blots are presented in Supplementary Fig. S6.

To determine the roles of EPAC2 in tube formation, we examined the effects of EPAC2 overexpression or knockdown on tube formation in TIME cells. Although EPAC2B and EPAC2C were increased during tube formation (Fig. 3b,c), only the effects of EPAC2B were investigated in this study because both proteins are responsive to cAMP due to their CNB-B domain. TIME cells were transduced by lentiviruses encoding EPAC2B or shRNAs for EPAC2, followed by sorting cells expressing GFP. EPAC2 expression was increased 25-fold in overexpressing cells (Fig. 4a) and decreased by 30% in knockdown cells (Fig. 4b). Because endogenous EPAC2 protein expression was hardly detectable due to the lower expression levels in the control state (Supplementary Fig. S3 online), EPAC2 knockdown was confirmed in EPAC2-overexpressing HEK293T cells (Fig. 4c). As shown in Fig. 4d,e, EPAC2 expression significantly affected tube formation in TIME cells. EPAC2 overexpression suppressed tube formation, as shown by the short tube length and large polygonal area (Fig. 4d). Alternatively, EPAC2 knockdown oppositely enhanced tube formation with a smaller polygonal area than the control (Fig. 4e). Additionally, we examined the effects of Forskolin, a drug that induces cAMP formation by activating adenylyl cyclase, on tube formation. It is reported that Forskolin induces Rap1 activation via EPAC in endothelial cells^11^. HMVECs on Matrigel containing Forskolin failed to form an appropriate mesh structure (Supplementary Fig. S5 online), suggesting that EPAC2 activation by cAMP is involved in tube formation via Rap1 activation. These results suggest that EPAC2 negatively regulates dense mesh organization during tube formation.

### EPAC2 affects cell morphology and migration in microvascular endothelial cells

**Figure 5:**
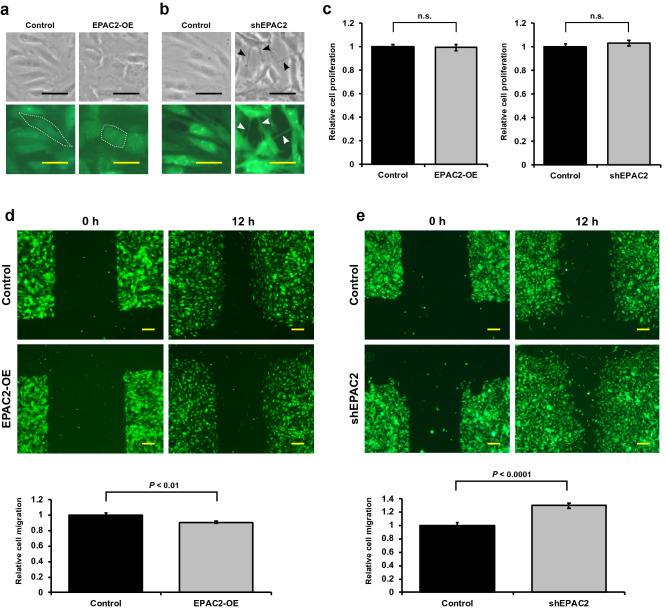
EPAC2 regulates cell migration of endothelial cells. (**a,b**) Cell morphology in EPAC2 overexpressing (**a**) and knockdown (**b**) TIME cells. Images were taken using phase contrast (upper panel) and fluorescence microscopy (lower panel). Representative cell shapes are surrounded by dotted lines. Filopodia-like protrusions are indicated with arrowheads. Scale bars: 50 μm. (**c**) Cell proliferation was evaluated as described in the Methods section. Data were expressed as mean ± S.E.M. (n = 6). (**d,e**) Cell migration of TIME cells was blocked by EPAC2 overexpression (**d**) and accelerated by EPAC2 knockdown (**e**). Cells were scratched and followed by culture for 12 h. Data were expressed as mean ± S.E.M. (d; n = 24 and e; n = 18). Scale bars: 200 μm.

Given that EPAC2 negatively affects the tube formation in microvascular endothelial cells, we examined the effect of EPAC2 expression on cell morphology. EPAC2 overexpression and knockdown led to a remarkable morphological change in TIME cells (Fig. 5a,b). Thus, EPAC2 overexpression likely changed endothelial cells to a round cell morphology (Fig. 5a). Furthermore, EPAC2 knockdown strikingly showed an elongated cell shape with filopodia-like protrusions like tip cells (Fig. 5b). To examine whether EPAC2 overexpression or knockdown affects cell proliferation, we measured cell numbers after two days of inoculation of monolayer cultures. As shown in Fig. 5c, neither EPAC2 overexpression nor EPAC2 knockdown changed the cell proliferation rate, suggesting that endothelial cell proliferation is unaffected by EPAC2. Alternatively, we measured migration distance using scratch assay (Fig. 5d,e). The distance between the cell edges was extended in EPAC2 overexpression (Fig. 5d) and narrowed in EPAC2 knockdown (Fig. 5e). These results suggest that EPAC2 prevents cell migration but not proliferation.

## Discussion

This study investigated the global gene expression changes in Matrigel-driven endothelial cell tubulogenesis and demonstrated that EPAC2 expression was strongly induced in tube-forming microvascular endothelial cells. Among the EPAC2 isoforms, EPAC2B and EPAC2C expressions were specifically increased during tube formation on Matrigel regardless of VEGF existence. EPAC2 suppressed endothelial cell migration and changed endothelial cell morphology, suggesting that EPAC2 acts as a negative regulator of an excessively formed network.

Our results also showed that MMP-10, HDAC9, and HECW2 were highly upregulated (Fig. 1b). It has been reported that MMP-10 is markedly induced during capillary tubular morphogenesis in the three-dimensional (3D) collagen model of vascular morphogenesis^19,20^. Endothelial cells may express these proteases for degrading the extracellular matrix to migrate on Matrigel and form a capillary-like structure. HDAC9 has been demonstrated to promote angiogenesis mediated by repressing of the miR-17-92 cluster^21^. The study showed that HDAC9 silencing decreased vessel formation in mice^21^. Furthermore, endothelial cell junctions were stabilized by the endothelial E3 ubiquitin ligase, HECW2, to promote angiogenesis^22^. HECW2 enhances AMOTL1 stability by lysine 63-linked ubiquitination to tighten cell-to-cell junctions^22^. The upregulation of these genes in our study suggested that an angiogenic status was reflected in Matrigel-driven tubulogenesis. In these conditions, we found the *RAPGEF4* gene which encodes EPAC2 to be upregulated in endothelial cell tubulogenesis for the first time.

Previous gene expression analyses have been performed by inducing capillary-like tubular structures into 3D fibrin matrix or on Matrigel in human umbilical vein endothelial cells or HMVECs^6–8,23^. Glesne et al. used Matrigel and HMVECs, but they reported the differential gene expression from 0.5 to 4 h post-Matrigel stimulation^7^. Meanwhile, we chose a time-point of 8 h. This time-point is suitable for investigating the gene expression profiling in the maturation of tubulogenesis since the network is completed at approximately 10 h and starts to break after 12 h. Our results demonstrated that EPAC2 expression was induced during tube formation and peaked at 6–8 h. The migration phase at the first 2 h on Matrigel showed the filopodia-like structure and low expression of EPAC2 in HMVECs. The signals from Matrigel and underexpression of EPAC2 may allow endothelial cells to differentiate into the tip cell-like phenotype. Consistent with this idea, the early phase of Matrigel-driven tubulogenesis (by 1 h post-plating) was reported to the increased expression of the tip cell marker, ﻿delta-like 4 homologue (Dll4)^7^. Therefore, if Matrigel directly stimulates EPAC2 expression, EPAC2 should be increased immediately after plating. However, EPAC2 expression was induced from 2 h on Matrigel, suggesting that the stimuli could be needed to induce EPAC2 expression in addition to Matrigel. During the reorganization and capillary formation by 8 h post-plating on Matrigel, EPAC2 expression was increased around eightfold compared with the control monolayer culture. Considering that EPAC2 suppressed dense mesh network formation in this study, the function of EPAC2 on tube formation could be the regulation of the proper network density. The tube formation with a proper dense network is thought to be executed by a negative regulator, such as EPAC2. Given the fact that EPAC2 inhibited endothelial cell migration and changed cells to a round morphology, EPAC2 expression could induce endothelial cells that compose capillary structures to lose tip cell-like characteristics and cell migratory capacities.

Although EPAC1 and EPAC2 act on the same downstream effectors, Rap1 and Rap2, their functions are mostly nonredundant because of distinct tissue and cellular distribution^9^. EPAC1 is mainly localized in the nuclear membrane and mitochondria^24^. The N-terminal domains are critical for the cellular localization of EPAC2^25^. EPAC2A, the longest isoform with a CNB-A domain (Fig. 3a), is localized near the plasma membrane, while EPAC2B is primarily present in the cytoplasm with a localization profile similar to EPAC1^25^. The localization of EPAC2C remains unknown. In this study, the EPAC2B and EPAC2C isoforms were induced on Matrigel. Furthermore, EPAC1 expression was not so much different between the control and tube formation, suggesting that EPAC2 acts as a regulator in endothelial cell tube formation. EPAC2B and EPAC2C are expressed mainly in the adrenal gland^14,25,26^ and liver, respectively, but these functions are largely unknown. EPAC2C has only CNB-B domain in regulatory region, but CNB-B region is sufficient to block the EPAC2 catalytic region^27^. This finding suggests that EPAC2C could be activated by cAMP as well as EPAC2B. We demonstrated that EPAC2B acts as a negative regulator of tubulogenesis (Figs. 4, 5); that is, EPAC2B suppresses endothelial cell migration and may prevent excessive tube formation. In contrast, EPAC2 knockdown enhanced cell migration with elongated cell shape, which stimulated tube formation. Consistent with our results, Hong et al. reported that EPAC2 and Rap1 are involved in endothelial cell morphology^28^. The study showed that anthrax edema toxin induces morphological change to a round shape and inhibits VEGF-induced migration but not proliferation in endothelial cells via EPAC2 and Rap1. Also, it has been reported that 8CPT-2Me-cAMP, an agonist for EPAC, induces endothelial actin rearrangement^11^. Therefore, EPAC2-Rap1 signaling could contribute to endothelial cell migration by inducing actin rearrangement, resulting in Matrigel-driven tubulogenesis suppression.

Although there are some limitations of the endothelial cell/Matrigel system to mimic the in vivo angiogenic processes, it shows many similar biological processes to in vivo angiogenesis, such as the formation of lumenized structures^5^. Concerning EPAC2, which we identified in this study, its function in in vivo angiogenesis has already been reported. EPACs and Rap1 have previously been demonstrated to inhibit angiogenesis in HMVECs^29^ and human dermal microvascular endothelial cells (HDMECs)^30^. One study showed that EPACs activation by 8CPT-2Me-cAMP and constitutive active Rap1 blocked the expression of an inhibitor of DNA binding 1 (Id1) in vivo^29^. Since Id1 suppresses the expression of thrombospondin-1 (TSP1) transcriptionally, EPACs and Rap1 activation increases TSP1 expression. TSP1 is reported to inhibit endothelial cell migration via CD36 or β_1_ integlin^31,32^, thus indicating the anti-angiogenic effects of EPACs and Rap1 activation^29^. Furthermore, EPAC2 activation by β-adrenoceptors decreased cell migration and tube formation in HDMECs and murine skin wound angiogenesis in vivo^30^. These reports support this study that EPAC2 changes cell morphology and inhibits cell migration in microvascular endothelial cells.

EPAC2 knockout (KO) mice have been generated, and KO mice have only subtle phenotype^33,34^. Some of these mice were generated by lacking a part of exon 1 and intron 1 of the mouse *Rapgef4* gene, resulting in the EPAC2A isoform-deficient mouse^16,33–36^. A study has made KO mouse by disrupting around exon 7, and consequently EPAC2A and EPAC2B deficiency^37^. It did not mention the status of the mouse and architecture of the vascular network, but KO mouse exhibited unaltered basal cardiac function. Kopperud et al. have generated EPAC2 KO mice by genomic deletion of exons 12–13 of *Rapgef4* gene^38^. The study demonstrated that the EPAC2 KO mouse was healthy and fertile and that EPAC2 was dispensable for the basal endothelial permeability without the description of the vascular abnormality. These findings indicate that EPAC2 has only a few contributions to mouse development. Similar to the mild phenotype of EPAC2 KO mice, Rap1a or Rap1b KO mice showed a normal phenotype unless the embryos died in utero^39,40^. However, Rap1 deficiency induced defective angiogenesis, endothelial cell migration, and tube formation^41–44^. Since Rap1 plays a vital role in cell adhesion and cell–cell junction^10,45^, endothelial cell function and angiogenesis are adequately affected by lacking Rap1. Considering that overactivation of Rap1 also inhibits angiogenesis as described above, Rap1 may act as both pro- and anti-angiogenic factors and need to be exquisitely regulated. Dramatic regulation of Rap1 may be unnecessary for maintaining endothelial cell function. Although the functions of endothelial cells in EPAC2 KO mice cannot be declared, EPAC2 may have only modest effects, which are mediated by Rap1, on endothelial cell functions. We demonstrated that tube formation and cell migration were significantly but slightly influenced by EPAC2 overexpression and knockdown in this study, supporting the idea that EPAC2 could finely tune angiogenesis via Rap1.

The angiogenic growth factor in the tube formation assay is normally supplemented by VEGF. Matrigel-driven tubulogenesis has not been performed without VEGF so far. However, we showed that a capillary-like structure was built on Matrigel without exogenous VEGF, suggesting that supplemented VEGF is not a driving force of tube formation. Alternatively, a capillary-like structure is not formed on the plate, even if VEGF is treated, indicating that the components of Matrigel are essential for tube formation. Extracellular matrices may be involved in tubulogenesis. Laminin, a major component of Matrigel, induces Dll4 expression and activates the tube formation via integrin in endothelial cells^46,47^, suggesting that laminin is a driving force of tube formation. The Dll4 expressing tip cell-like cells induce the differentiation into the stalk cell-like phenotype in neighbors via Dll4-Notch signaling. The control of Notch signaling via Dll4 is involved in the control of tip-stalk cell balance^2^. In this study, EPAC2 isoforms but not EPAC1 were specifically induced during tube formation. EPAC2 isoform expression in different tissues was reported to be regulated by DNA methylation of alternative promoters^26^. However, the mechanism of the transcriptional regulation of EPAC2 expression is unknown.

Given that EPAC2 expression was stimulated 2 h after but not just after plating, laminin-integrin signaling is not a direct upstream factor for EPAC2 expression. Instead, the differentiated tip cell- or stalk cell-like cells may produce some factors that induce EPAC2 expression in tip cell-like cells to prevent the formation of the protrusions and cell migration possibly via actin rearrangement as described above. The promoter region of EPAC2B is in intron 4 of the *RAPGEF4* gene, and the alternative exon 1b is used for exon 1 of EPAC2B. On the other hand, EPAC2C uses different transcriptional start points in intron 9 of the *RAPGEF4* gene and commences translation at the end of exon 10, resulting in the lack of both CNB-A and DEP domains of EPAC2A. It is unclear what *cis*-elements and regulatory factors govern the specific expression of EPAC2B and EPAC2C, but this needs to be further elucidated.

This study indicated for the first time that EPAC2 is involved as a negative regulator in the capillary-like tube formation of microvascular endothelial cells. Although additional studies are needed to clarify the function of EPAC2 in tubulogenesis, these findings have revealed new regulatory features in vascular endothelial cell tube formation. The results obtained in this study may provide new clues to clarify further the mechanisms in regulating capillary-like tube formation and angiogenesis, especially in the proper network formation of vascular tube formation, and for developing therapeutic strategies to treat angiogenesis-related diseases.

## Methods

### Cell culture

HMVECs (Cascade Biologics Inc.) and TIME cells (#CRL4025, ATCC) were maintained in HuMedia-EB2 (HMEB2) medium supplemented with 5% FBS, 5 ng/mL basic fibroblast growth factor, 10 μg/mL heparin, 10 ng/mL epidermal growth factor, 1 μg/mL hydrocortisone, 39.3 μg/mL (80 µM) dibutyryl cAMP, 50 µg/mL gentamicin and 50 ng/mL amphotericin B (growth medium) according to the manufacturer’s instructions (Kurabo Corp., Tokyo Japan). HEK293TN cells (System Biosciences) were maintained in DMEM (Sigma-Aldrich) supplemented with 10% FBS, 100 IU/mL penicillin, and 100 µg/mL streptomycin.

### Microarray

The microarray analysis was performed as previously described^48^. Briefly, cells were cultured on Matrigel for 8 h in a 100-mm plate and washed with 15 mM HEPES/PBS, followed by Matrigel digestion with dispase for 10 min at 37 °C. Cells were lysed with TRIzol and purified using RNeasy Plus mini kit (Qiagen). The RNA quality was determined by the 28S/18S ratios of ribosomal RNA band intensities on electrophoresis gels under denaturation. Subsequently, 300 ng of total RNA was labeled according to the manufacturer’s instructions for the GeneChip WT Sense Target Labeling Kit (Affymetrix). Fragmented and labeled cDNAs were then hybridized onto the Affymetrix GeneChip Human Gene 1.0 ST arrays in a GeneChip Hybridization Oven 640 (Affymetrix). Arrays were washed and stained using the GeneChip Fluidics Station 450 and detected using a 3000 7G GeneChip Scanner (Affymetrix). All arrays passed the quality control criteria of the Expression Console software (Affymetrix). Raw data CEL files were then normalized using the RMA algorithm, and the data were exported using Expression Console or Gene Spring software version 10.0.1 (Agilent Technologies).

### RT-qPCR

Total RNA was extracted from cells using RNeasy Mini kits (Qiagen). Subsequently, 0.1–0.6 µg aliquots of total RNA samples were reverse transcribed using High-Capacity cDNA Reverse Transcription Kits (Thermo Fisher Scientific). qPCR was performed using a StepOnePlus Real-Time System (Thermo Fisher Scientific). EPAC2 mRNA levels were measured using TaqMan Gene Expression Assay (Thermo Fisher Scientific: Hs00199754_m1 or Hs00899815_m1). Predeveloped TaqMan Assay Reagent Control kits (Human GAPDH or Human ACTB Endogenous Control, Thermo Fisher Scientific) were used as internal controls.

### RT-PCR

RT-PCR was performed by RT reaction at 55 °C for 30 min, followed by adequate cycles at 94 °C for 15 s, 60 °C for 30 s, and 68 °C for 30 s using a SuperScript III One-Step RT-PCR System with Platinum *Taq* DNA Polymerase (Thermo Fisher Scientific) (primers are listed in Table 1). The amounts of RNA and amplification cycle numbers were determined from linear amplification kinetics. DNA band intensity was analyzed and quantified by ImageJ (http://imagej.nih.gov/ij/).

### Lentivirus production and transduction

The coding region of EPAC2B isoform (NM_001100397.2) was amplified by RT-PCR using the Prime Script High Fidelity RT-PCR kit (TaKaRa) and cloned into Nhe I and Not I sites of the pCDH-CMV-MCS-EF1-copGFP vector (System Biosciences). shRNA vectors were constructed using the pSIH-H1-copGFP shRNA vector (System Biosciences) by inserting synthetic oligonucleotides listed in Table 1. Pseudoviral particles were generated by co-transfecting the pCDH vector or pSIH vector and pPACKH1 packaging plasmid mix (System Biosciences) into HEK293TN cells in a 6-well plate using Lipofectamine and Plus Reagent (Life Technologies). A 72 h cultured media supernatant was collected and filtered through a Millex HV 0.45 µm PVDF membrane. Pseudoviral particles were then transduced into TIME cells seeded at a density of 2 × 10^5^ cells per well in a 6-well plate. After transduction, higher GFP expressing TIME cells were sorted by GFP intensity using a SH800 Cell Sorter (Sony) and used for experiments.

### Western blotting

Cells were suspended in Laemmli-sample buffer containing 0.2 µM DTT and shredded using a QIA shredder (Qiagen) and heated at 98 °C for 5 min. The resulting protein samples were applied to SDS-PAGE using 7.5% polyacrylamide gel (ATTO). After electrophoresis, proteins were transferred onto PVDF membranes (ATTO) and blocked with 5% skim milk in 0.1% Tween 20/PBS (TPBS). Anti-EPAC2 (5B1, Cell Signaling Technology) or anti-β-actin (Sigma-Aldrich) antibodies in 1% BSA/TPBS were used as primary antibodies, and anti-mouse IgG-HRP (Cell Signaling Technology) or anti-rabbit IgG-HRP (GE Healthcare) in 1% BSA/TPBS were used as secondary antibodies. Protein signals were detected using an ECL Plus Chemiluminescent Substrate (Thermo Fisher Scientific) using Molecular Dynamics Typhoon 9400 Imager.

### Tube formation assay

The tube formation assay was performed as previously described^49,50^. Briefly, growth factor-reduced Matrigel (BD Biosciences) were prepared at the bottom chambers of µ-Slide Angiogenesis ibiTreat (ibidi). 5000 cells of HMVECs and TIME cells in HMEB2 supplemented with 0.5% FBS were added on Matrigel in the presence or absence of 30 ng/mL VEGF_165_ (Millipore). Tube structure was observed using ZEISS Axiovert A1 microscope (Zeiss). Tube length and the polygonal area were measured using ImageJ. The positions of sprawling and lined cells were over-drawn with straight lines. The sum of the line lengths from one image in a single well, and then the mean of the sum of tube length from ten images was calculated as tube length. The polygonal area was measured as the mean of the area surrounded by the straight lines from one image, and then the average of the mean of the polygonal area from ten images was calculated.

### Cell migration assay

Lentiviral-transduced TIME cells were seeded at a density of 5 × 10^4^ cells per well in a 12-well plate in 1.6 ml of the growth medium. After 4 d, confluent cell layers were scratched and changed to the new growth medium (0 h), and were incubated for 12 h. The distances between one side of the scratch and the other were measured at 0 and 12 h. Cell migration was calculated by subtracting the distance at 0 h from a distance at 12 h.

### Cell proliferation assay

Cell proliferation assay was performed as previously described^51^. Briefly, lentiviral-transduced TIME cells were seeded at a density of 3 × 10^3^ cells per well in a 96-well plate in 0.2 mL of the growth medium. The cells were incubated for 1 d or 3 d at 37 ºC. After incubation, cell proliferation was assessed by cellular reduction of the tetrazolium salt, WST-8, to formazan (Cell Counting Kit-8, Dojindo Laboratories). The ratio of absorbance (450 nm) of 1 d to 3 d was calculated as the cell proliferation.

### Statistical analysis

Statistical significance of the data was determined using Student’s t-test for unpaired data. Multiple comparisons were analyzed by one-way ANOVA Tukey–Kramer test using the R package (version 3.5.1; https://www.r-project.org).

## Supplementary Information

Supplementary Information.
