# Supplementary material for: EPAC2 acts as a negative regulator in Matrigel-driven tubulogenesis of human microvascular endothelial cells
Source: Sci Rep. 2021 Sep 30;11:19453. doi: 10.1038/s41598-021-98906-9 (PMC8484440; doi:10.1038/s41598-021-98906-9)
Supplement: Supplementary file 1 — Supplementary Information. [file 41598_2021_98906_MOESM1_ESM.pdf]

Supplementary Information

EPAC2 acts as a negative regulator in Matrigel-driven tubulogenesis of human microvascular endothelial cells

Takayuki Ikeda[1,3,*], Yoshino Yoshitake[1,3], Yasuo Yoshitomi[1], Hidehito Saito-Takatsuji[1], Yasuhito Ishigaki[2] & Hideto Yonekura[1]

[1]Department of Biochemistry, Kanazawa Medical University School of Medicine, 1-1 Daigaku, Uchinada, Kahoku-gun, Ishikawa 920-0293, Japan. [2]Division of Molecular and Cell Biology, Medical Research Institute, Kanazawa Medical University, 1-1 Daigaku, Uchinada, Kahoku-gun, Ishikawa 920-0293, Japan. [3]Takayuki Ikeda and Yoshino Yoshitake contributed equally to this study. [*]email: tikeda@kanazawa-med.ac.jp

Supplementary Table S1. The microarray data.

[Exp1, 0 h](raw)	[Exp1, 8h](raw)	Fold change1	[Exp2, 0 h](raw)	[Exp2, 8 h](raw)	Fold change2	Av Fold change	gene description	genbank	genesymbol	Location	Type	refseq	unigene
124.6	455.9	3.658	183.3	1740.3	9.495	6.576	matrix metallopeptidase 10 (stromelysin 2)	AK222601	MMP10	Extracellular Space	peptidase	NM_002425	Hs.2258///Hs.2258///Hs.2258
210.8	1660.8	7.877	399.7	1564.5	3.914	5.896	Rap guanine nucleotide exchange factor (GEF) 4	AB209681	RAPGEF4	Cytoplasm	other	NM_007023|NM_001100397	Hs.470646///Hs.470646///Hs.470646///Hs.470646///Hs.470646///Hs.470646///Hs.470646///Hs.4
85.6	646.3	7.549	98.9	404.5	4.089	5.819	histone deacetylase 9	AY197371	HDAC9	Nucleus	transcription regulator	NM_178423|NM_178425|NM_058176|NM_058177|NM_014707	Hs.196054///Hs.196054///Hs.196054///Hs.196054///Hs.196054///Hs.196054///Hs.196054///Hs.196054
1829.2	2968.0	1.623	105.3	917.6	8.716	5.170	matrix metallopeptidase 1 (interstitial collagenase)	M13509	MMP1	Extracellular Space	peptidase	NM_002421	Hs.83169///Hs.83169///Hs.83169
140.6	408.2	2.905	191.8	1393.0	7.261	5.083	potassium intermediate/small conductance calcium-activated channel, subfamily N, member 2	AF239613	KCNN2	Plasma Membrane	ion channel	NM_021614	Hs.98280///Hs.98280///Hs.98280///Hs.98280
656.2	3369.6	5.135	654.7	2946.3	4.500	4.817	HECT, C2 and WW domain containing E3 ubiquitin protein ligase 2	BC117194	HECW2	Unknown	enzyme	NM_020760	Hs.654742///Hs.654742///Hs.654742
61.5	312.4	5.076	81.1	283.4	3.495	4.285							
495.7	2084.0	4.204	310.3	1316.9	4.244	4.224	solute carrier family 7, (cationic amino acid transporter, y+ system) member 11	AF252872	SLC7A11	Plasma Membrane	transporter	NM_014331	Hs.390594///Hs.390594///Hs.390594
250.3	938.3	3.749	169.0	782.3	4.630	4.189	neuronal pentraxin I	U61849	NPTX1	Extracellular Space	other	NM_002522	Hs.514556///Hs.702002///Hs.514556///Hs.702002///Hs.514556///Hs.702002
115.7	205.5	1.777	298.7	1869.3	6.259	4.018	guanylate cyclase 1, soluble, alpha 3	BX647200	GUCY1A3	Cytoplasm	enzyme	NM_000856	Hs.24258///Hs.24258///Hs.24258///Hs.24258///Hs.24258
439.8	1779.3	4.045	513.3	1899.3	3.700	3.873	Cas-Br-M (murine) ecotropic retroviral transforming sequence b	BC032851	CBLB	Nucleus	other	NM_170662	Hs.430589///Hs.430589///Hs.430589///Hs.430589///Hs.430589
58.9	168.6	2.864	137.6	661.9	4.809	3.836	phospholipase A2, group IVA (cytosolic, calcium-dependent)	AK290336	PLA2G4A	Cytoplasm	enzyme	NM_024420	Hs.497200///Hs.497200///Hs.497200
417.5	1398.9	3.351	386.9	1611.4	4.165	3.758	ATPase, class I, type 8B, member 1	AF038007	ATP8B1	Plasma Membrane	transporter	NM_005603	Hs.216623///Hs.216623///Hs.216623
61.9	162.4	2.623	80.0	387.5	4.845	3.734							
336.3	1266.0	3.764	372.4	1335.4	3.586	3.675	zinc finger CCCH-type containing 12C	AB096241	ZC3H12C	Unknown	other	NM_033390	Hs.376289///Hs.376289///Hs.376289
116.9	626.2	5.357	98.8	176.2	1.783	3.570	nuclear receptor subfamily 5, group A, member 2	AB019246	NR5A2	Nucleus	ligand-dependent nuclear receptor	NM_205860|NM_003822	Hs.33446///Hs.33446///Hs.33446///Hs.33446///Hs.33446///Hs.33446///Hs.33446
127.6	516.2	4.047	123.8	382.5	3.089	3.568	Rho family GTPase 1	BC026356	RND1	Cytoplasm	enzyme	NM_014470	Hs.124940///Hs.124940///Hs.124940
281.0	1030.0	3.666	285.4	960.5	3.366	3.516	diacylglycerol kinase, delta 130kDa	AB078966	DGKD	Cytoplasm	kinase	NM_152879|NM_003648	Hs.471675///Hs.471675///Hs.471675///Hs.471675
1363.4	5205.8	3.818	1305.4	4082.1	3.127	3.473	solute carrier family 12 (sodium/potassium/chloride transporters), member 2	AY280459	SLC12A2	Plasma Membrane	transporter	NM_001046	Hs.162585///Hs.162585///Hs.162585///Hs.162585
179.5	911.3	5.077	146.3	272.3	1.862	3.469	ADAM metallopeptidase with thrombospondin type 1 motif, 1	BC036515	ADAMTS1	Extracellular Space	peptidase	NM_006988	Hs.643357///Hs.643357///Hs.643357///Hs.643357
202.3	424.7	2.099	106.3	509.2	4.793	3.446	guanylate binding protein 2, interferon-inducible	BC073163	GBP2	Cytoplasm	enzyme	NM_004120	Hs.386567///Hs.386567///Hs.386567
280.8	892.7	3.179	183.1	659.6	3.603	3.391	ring finger protein 122	AK022588	RNF122	Unknown	other	NM_024787	Hs.151237///Hs.151237///Hs.151237
567.7	1660.4	2.925	281.7	1048.3	3.722	3.323	solute carrier family 6 (neurotransmitter transporter, taurine), member 6	U16120	SLC6A6	Plasma Membrane	transporter	NM_003043	Hs.529488///Hs.529488///Hs.529488
1384.0	2355.7	1.702	324.5	1586.9	4.890	3.296	neuropilin 1	AK292822	NRP1	Plasma Membrane	transmembrane receptor	NM_003873|NM_001024628|NM_001024629	Hs.131704///Hs.131704///Hs.131704///Hs.131704///Hs.131704///Hs.131704///Hs.131704///Hs.131704///Hs.131704///Hs.131704///Hs.131704///Hs.131704///Hs.131704///Hs.131704///Hs.13170
77.3	261.9	3.389	100.9	318.1	3.151	3.270	ankyrin repeat domain 44	BC016985	ANKRD44	Unknown	other	NM_153697	Hs.712566///Hs.712566///Hs.712566///Hs.712566///Hs.712566
833.7	2240.0	2.687	267.6	1021.0	3.815	3.251	fms-related tyrosine kinase 1 (vascular endothelial growth factor/vascular permeability factor receptor)	AF063657	FLT1	Plasma Membrane	kinase	NM_002019	Hs.654360///Hs.654360///Hs.654360
143.2	356.0	2.486	98.6	395.6	4.013	3.249	dual specificity phosphatase 10	BC031405	DUSP10	Nucleus	phosphatase	NM_007207|NM_144728|NM_144729	Hs.497822///Hs.497822///Hs.497822///Hs.497822///Hs.497822///Hs.497822///Hs.497822///Hs.4
583.7	1192.2	2.042	247.6	1029.2	4.157	3.100	serpin peptidase inhibitor, clade E (nexin, plasminogen activator inhibitor type 1), member 2	BC042628	SERPINE2	Extracellular Space	other	NM_006216	Hs.38449///Hs.38449///Hs.38449
221.2	809.7	3.660	66.5	168.7	2.537	3.099	KIT ligand	BC126166	KITLG	Extracellular Space	growth factor	NM_000899|NM_003994	Hs.1048///Hs.1048///Hs.1048///Hs.1048///Hs.1048///Hs.1048
215.1	821.3	3.819	209.8	486.3	2.318	3.068	Meis homeobox 2	BC007202	MEIS2	Nucleus	transcription regulator	NM_172316|NM_170677|NM_170674|NM_002399|NM_172315|NM_170675|NM_17	Hs.510989///Hs.510989///Hs.510989///Hs.510989///Hs.510989///Hs.510989///Hs.510989///Hs.510989///Hs.510989///Hs.510989///Hs.510989///Hs.510989///Hs.510989///Hs.510989///Hs.51098
278.7	811.7	2.912	264.9	847.7	3.200	3.056	Rho guanine nucleotide exchange factor (GEF) 3	AF433662	ARHGEF3	Cytoplasm	other	NM_019555	Hs.476402///Hs.476402///Hs.476402///Hs.476402
171.7	590.1	3.437	132.0	349.1	2.645	3.041	tripartite motif-containing 2	AF220018	TRIM2	Cytoplasm	other	NM_015271	Hs.435711///Hs.435711///Hs.435711
248.5	782.7	3.150	238.4	681.0	2.856	3.003	sprouty homolog 4 (Drosophila)	AF227516	SPRY4	Plasma Membrane	other	NM_030964	Hs.323308///Hs.323308///Hs.323308
82.6	213.2	2.582	254.0	849.6	3.345	2.963	sema domain, immunoglobulin domain (Ig), short basic domain, secreted, (semaphorin) 3D	AY358937	SEMA3D	Extracellular Space	other	NM_152754	Hs.201340///Hs.201340///Hs.201340
396.2	1237.0	3.122	188.2	511.0	2.716	2.919	PR domain containing 1, with ZNF domain	AY198414	PRDM1	Nucleus	transcription regulator	NM_001198|NM_182907	Hs.436023///Hs.436023///Hs.436023///Hs.436023///Hs.436023///Hs.436023///Hs.436023
351.3	1234.1	3.513	318.0	735.7	2.313	2.913	ST8 alpha-N-acetyl-neuraminide alpha-2,8-sialyltransferase 4	BC053657	ST8SIA4	Cytoplasm	enzyme	NM_005668|NM_175052	Hs.308628///Hs.308628///Hs.308628///Hs.308628
677.2	1134.2	1.675	448.2	1829.7	4.083	2.879	E2F transcription factor 7	BC017481	E2F7	Nucleus	transcription regulator	NM_203394	Hs.416375///Hs.416375///Hs.416375///Hs.416375
158.1	466.7	2.952	276.3	766.0	2.772	2.862	tumor necrosis factor, alpha-induced protein 3	BC064689	TNFAIP3	Nucleus	other	NM_006290	Hs.591338///Hs.591338///Hs.591338
566.8	2045.3	3.608	919.0	1882.2	2.048	2.828		AK289737	KIAA1946	Unknown	other	NM_177454	Hs.28872///Hs.28872///Hs.28872///Hs.28872
545.2	1852.2	3.397	738.9	1636.3	2.214	2.806	ATPase, Ca++ transporting, plasma membrane 1	J04027	ATP2B1	Plasma Membrane	transporter	NM_001001323|NM_001682	Hs.506276///Hs.506276///Hs.506276///Hs.506276///Hs.506276///Hs.506276///Hs.506276
490.5	1745.7	3.559	650.8	1196.4	1.838	2.699	heme oxygenase (decycling) 1	BC001491	HMOX1	Cytoplasm	enzyme	NM_002133	Hs.517581///Hs.517581///Hs.517581
112.4	269.3	2.396	86.0	256.6	2.982	2.689		AL832451					
596.5	2207.7	3.701	1504.0	2512.0	1.670	2.685	matrix Gla protein	BC093078	MGP	Extracellular Space	other	NM_000900	Hs.365706///Hs.365706///Hs.365706
177.6	359.4	2.023	145.1	470.4	3.241	2.632	coagulation factor III (thromboplastin, tissue factor)	J02931	F3	Plasma Membrane	transmembrane receptor	NM_001993	Hs.62192///Hs.62192///Hs.62192///Hs.62192
131.6	365.8	2.779	146.1	360.0	2.463	2.621	EFR3 homolog B (S. cerevisiae)	BC049384|AK095190	EFR3B	Unknown	other		Hs.654648///Hs.654648///Hs.654648///Hs.654648
309.9	810.2	2.615	261.7	684.1	2.614	2.614		BC050537	FLJ20160	Unknown	other	NM_017694	Hs.418581///Hs.708489///Hs.418581///Hs.708489///Hs.418581///Hs.708489///Hs.418581///Hs.7
293.5	504.7	1.720	178.9	618.5	3.458	2.589	G-protein signaling modulator 2 (AGS3-like, C. elegans)	BC027732	GPSM2	Nucleus	other	NM_013296	Hs.584901///Hs.584901///Hs.584901///Hs.584901
107.7	270.9	2.515	104.2	271.7	2.607	2.561	chemokine (C-C motif) ligand 5	M21121	CCL5	Extracellular Space	cytokine	NM_002985	Hs.514821///Hs.514821///Hs.514821
711.6	1556.9	2.188	449.2	1280.3	2.850	2.519	dual specificity phosphatase 5	BC062545	DUSP5	Nucleus	phosphatase	NM_004419	Hs.2128///Hs.2128///Hs.2128
401.7	858.4	2.137	230.4	655.8	2.847	2.492	epidermal growth factor receptor pathway substrate 8	BC030010	EPS8	Plasma Membrane	peptidase	NM_004447	Hs.591160///Hs.591160///Hs.591160
624.1	1490.0	2.387	547.6	1419.3	2.592	2.490	heparin-binding EGF-like growth factor	BC033097	HBEGF	Extracellular Space	growth factor	NM_001945	Hs.799///Hs.799///Hs.799
299.7	757.2	2.527	401.5	977.1	2.434	2.480	transmembrane protein 140	BC020942	TMEM140	Unknown	other	NM_018295	Hs.658637///Hs.658637///Hs.658637
655.8	1210.2	1.845	278.5	860.7	3.091	2.468	mal, T-cell differentiation protein-like	AK125647	MALL	Plasma Membrane	other	NM_005434	Hs.185055///Hs.185055///Hs.185055
389.4	1101.1	2.828	419.2	876.6	2.091	2.460	inositol polyphosphate-5-phosphatase, 40kDa	BC096280	INPP5A	Plasma Membrane	phosphatase	NM_005539	Hs.709221///Hs.709221///Hs.709221///Hs.709221///Hs.709221///Hs.709221
647.3	1482.2	2.290	1097.8	2770.9	2.524	2.407	GRB2-associated binding protein 1	BC064848	GAB1	Cytoplasm	other	NM_207123|NM_002039	Hs.80720///Hs.618456///Hs.80720///Hs.618456///Hs.80720///Hs.618456///Hs.80720///Hs.61845
136.3	309.7	2.273	334.0	846.5	2.535	2.404	CNKSR family member 3	AY328891	CNKSR3	Plasma Membrane	other	NM_173515	Hs.16064///Hs.16064///Hs.16064///Hs.16064///Hs.16064
171.6	530.2	3.089	405.5	690.6	1.703	2.396	platelet derived growth factor D	BC030645	PDGFD	Extracellular Space	growth factor	NM_025208|NM_033135	Hs.352298///Hs.352298///Hs.352298///Hs.352298///Hs.352298
204.9	599.1	2.924	426.6	788.8	1.849	2.387	neural precursor cell expressed, developmentally down-regulated 4-like	AB071179	NEDD4L	Cytoplasm	enzyme	NM_015277	Hs.185677///Hs.185677///Hs.185677///Hs.185677///Hs.185677///Hs.185677///Hs.185677///Hs.1
2064.0	6125.3	2.968	3534.6	6277.3	1.776	2.372	calcitonin receptor-like	U17473|DQ280491	CALCRL	Plasma Membrane	G-protein coupled receptor	NM_005795	Hs.470882///Hs.470882///Hs.470882///Hs.470882
972.0	2403.9	2.473	495.4	1124.0	2.269	2.371	B-cell CLL/lymphoma 6, member B (zinc finger protein)	BC059404	BCL6B	Nucleus	transcription regulator	NM_181844	Hs.22575///Hs.22575///Hs.22575
469.2	913.8	1.948	389.3	1080.3	2.775	2.361	chromosome 1 open reading frame 21	AF312864	C1orf21	Unknown	other	NM_030806	Hs.497159///Hs.571795///Hs.497159///Hs.571795///Hs.497159///Hs.571795///Hs.497159///Hs.5
200.4	620.2	3.094	190.7	308.7	1.619	2.357	intercellular adhesion molecule 1 (CD54), human rhinovirus receptor	BC015969	ICAM1	Plasma Membrane	transmembrane receptor	NM_000201	Hs.707983///Hs.707983///Hs.707983
77.5	195.1	2.517	102.7	217.7	2.121	2.319	NIMA (never in mitosis gene a)- related kinase 10	AK123061	NEK10	Unknown	kinase	NM_001031741|NM_152534	Hs.506115///Hs.506115///Hs.506115///Hs.506115///Hs.506115///Hs.506115///Hs.506115///Hs.5
194.0	334.3	1.723	159.2	455.5	2.861	2.292	FERM domain containing 3	EF560742	FRMD3	Unknown	other	NM_174938	Hs.709357///Hs.709357///Hs.709357///Hs.709357///Hs.709357///Hs.709357///Hs.709357
61.1	154.1	2.520	50.8	104.6	2.059	2.289	GULP, engulfment adaptor PTB domain containing 1	AK055718	GULP1	Cytoplasm	other	NM_016315	Hs.470887///Hs.470887///Hs.470887
179.3	373.6	2.083	204.4	508.2	2.487	2.285	nuclear factor, interleukin 3 regulated	U26173	NFIL3	Nucleus	transcription regulator	NM_005384	Hs.79334///Hs.79334///Hs.79334///Hs.79334
24.8	49.7	2.003	27.0	69.2	2.562	2.282		AK123971					
504.5	1015.2	2.012	465.2	1172.4	2.520	2.266	transmembrane protein 47	BC039242	TMEM47	Plasma Membrane	other	NM_031442	Hs.8769///Hs.8769///Hs.8769
34.2	61.0	1.781	65.5	179.5	2.741	2.261	CMT1A duplicated region transcript 1 | F-box and WD repeat domain containing 10 | tripartite motif-containing 16	BC028364|AB209899	CDRT1|FBXW10|TRIM16	Unknown	other	NM_006382	Hs.548021///Hs.632233///Hs.592128///Hs.592128///Hs.123534///Hs.548021///Hs.632233///Hs.123534///Hs.548021///Hs.632233///Hs.123534
1963.2	3488.2	1.777	1126.8	3080.5	2.734	2.255	G protein-coupled receptor 177	AY359035	GPR177			NM_024911|NM_001002292	Hs.647659///Hs.647659///Hs.647659///Hs.647659///Hs.647659///Hs.647659///Hs.647659///Hs.6
1481.3	3284.3	2.217	1541.0	3531.3	2.292	2.254	sperm specific antigen 2	AB067514	SSFA2	Plasma Membrane	other	NM_006751	Hs.591602///Hs.591602///Hs.591602///Hs.591602
276.2	445.6	1.613	246.5	709.3	2.878	2.245	chromosome 14 open reading frame 139	BC008299|CR457337	C14orf139	Unknown	other		Hs.41502///Hs.41502///Hs.41502
76.0	127.7	1.680	73.1	204.0	2.790	2.235	interferon regulatory factor 6	BC014852	IRF6	Nucleus	transcription regulator	NM_006147	Hs.591415///Hs.591415///Hs.591415
1323.5	3098.9	2.341	1236.1	2631.0	2.128	2.235	N-myc downstream regulated gene 1	AK126924	NDRG1	Nucleus	kinase	NM_006096	Hs.372914///Hs.618002///Hs.372914///Hs.618002///Hs.372914///Hs.618002///Hs.372914///Hs.6
2233.3	4356.6	1.951	2318.9	5702.6	2.459	2.205	arrestin domain containing 3	BC053619	ARRDC3	Unknown	other	NM_020801	Hs.24684///Hs.24684///Hs.24684
228.3	569.3	2.493	169.9	322.7	1.899	2.196	retinoic acid receptor, gamma	AK290588	RARG	Nucleus	ligand-dependent nuclear receptor	NM_000966|NM_001042728	Hs.1497///Hs.1497///Hs.1497///Hs.1497///Hs.1497///Hs.1497
301.6	582.1	1.930	246.3	598.0	2.428	2.179	coiled-coil domain containing 88C	AF070587	CCDC88C	Unknown	other	NM_001080414	Hs.525536///Hs.525536///Hs.525536///Hs.525536
337.0	661.3	1.962	427.1	1022.9	2.395	2.179	regulator of calcineurin 1	AY325903	RCAN1	Nucleus	transcription regulator	NM_004414|NM_203417|NM_203418	Hs.282326///Hs.282326///Hs.282326///Hs.282326///Hs.282326///Hs.282326///Hs.282326///Hs.2
863.1	1961.8	2.273	487.9	1012.2	2.074	2.174	solute carrier family 9 (sodium/hydrogen exchanger), member 3 regulator 2	BC014513	SLC9A3R2	Plasma Membrane	transporter	NM_004785	Hs.440896///Hs.440896///Hs.440896
318.2	642.8	2.020	446.1	1028.7	2.306	2.163	phospholipase C, beta 1 (phosphoinositide-specific)	AB011153	PLCB1	Cytoplasm	enzyme	NM_182734|NM_015192	Hs.431173///Hs.431173///Hs.431173///Hs.431173///Hs.431173///Hs.431173///Hs.431173
714.8	1727.5	2.417	596.3	1137.4	1.907	2.162	Cbp/p300-interacting transactivator, with Glu/Asp-rich carboxy-terminal domain, 2	BC004377	CITED2	Nucleus	transcription regulator	NM_006079	Hs.82071///Hs.82071///Hs.82071///Hs.82071
809.8	1565.1	1.933	763.1	1812.9	2.376	2.154	tripartite motif-containing 25	BC042541	TRIM25	Cytoplasm	transcription regulator	NM_005082	Hs.528952///Hs.528952///Hs.528952///Hs.528952
333.3	687.3	2.062	402.7	903.4	2.244	2.153	brain protein 44-like	BC000810	BRP44L	Cytoplasm	other	NM_016098	Hs.172755///Hs.172755///Hs.172755///Hs.172755///Hs.172755///Hs.172755
518.4	1133.8	2.187	758.2	1605.2	2.117	2.152	solute carrier family 12 (potassium/chloride transporters), member 6	DQ138323	SLC12A6	Plasma Membrane	transporter	NM_001042496|NM_133647|NM_001042494|NM_001042495|NM_001042497|NM_0	Hs.510939///Hs.510939///Hs.510939///Hs.510939///Hs.510939///Hs.510939///Hs.510939///Hs.510939///Hs.510939///Hs.510939///Hs.510939

69.2	116.3	1.680	95.1	249.0	2.618	2.149	titin	BX537998	TTN	Cytoplasm	peptidase	NM_133378|NM_133437|NM_133432|NM_003319|NM_133379	Hs.654592///Hs.654592///Hs.654592///Hs.654592///Hs.654592///Hs.654592///Hs.654592///Hs.654592///Hs.654592///Hs.654592///Hs.654592///Hs.654592///Hs.654592///Hs.654592
165.7	313.0	1.889	90.2	212.7	2.359	2.124	phosphate regulating endopeptidase homolog, X-linked (hypophosphatemia, vitamin D resistant rickets)	U82970	PHEX	Cytoplasm	peptidase	NM_000444	Hs.495834///Hs.495834///Hs.495834
148.8	291.7	1.961	215.4	492.5	2.286	2.124							
1170.3	2015.7	1.722	768.5	1937.6	2.521	2.122	SMAD family member 3	BC050743	SMAD3	Nucleus	transcription regulator	NM_005902	Hs.618504///Hs.702172///Hs.618504///Hs.702172///Hs.618504///Hs.702172
543.6	1112.2	2.046	708.7	1536.5	2.168	2.107	tumor necrosis factor (ligand) superfamily, member 18	AY358868	TNFSF18	Extracellular Space	cytokine	NM_005092	Hs.248197///Hs.248197///Hs.248197
144.3	345.5	2.395	283.6	514.9	1.816	2.105		AF448858|AK095297	LOC284702	Unknown	other		Hs.657313///Hs.657313
519.1	898.6	1.731	180.0	444.4	2.468	2.100	ADAM metallopeptidase with thrombospondin type 1 motif, 4	BC063293	ADAMTS4	Extracellular Space	peptidase	NM_005099	Hs.211604///Hs.211604///Hs.211604///Hs.211604
1452.4	3308.6	2.278	844.4	1621.1	1.920	2.099	alpha-2-macroglobulin	BC040071	A2M	Extracellular Space	transporter	NM_000014	Hs.212838///Hs.212838///Hs.212838
44.3	78.5	1.771	56.4	136.7	2.426	2.099	chromosome 7 open reading frame 53	BC031976	C7orf53	Unknown	other	NM_182597	Hs.396189///Hs.396189///Hs.396189
596.9	1073.8	1.799	493.5	1172.6	2.376	2.087	vacuolar protein sorting 37 homolog B (S. cerevisiae)	BC005882	VPS37B	Nucleus	other	NM_024667	Hs.507162///Hs.507162///Hs.507162
978.6	2240.6	2.290	1443.0	2699.9	1.871	2.080	sperm associated antigen 9	BC146755	SPAG9	Plasma Membrane	other	NM_003971	Hs.463439///Hs.593620///Hs.463439///Hs.593620///Hs.463439///Hs.593620///Hs.463439///Hs.593620///Hs.463439///Hs.593620///Hs.463439///Hs.593620///Hs.463439///Hs.593620
467.0	840.6	1.800	542.2	1274.3	2.350	2.075	plasminogen activator, tissue	BC095403	PLAT	Extracellular Space	peptidase	NM_000930|NM_033011	Hs.491582///Hs.491582///Hs.491582///Hs.491582///Hs.491582///Hs.491582///Hs.491582
539.6	1337.8	2.479	575.0	953.2	1.658	2.068	neural precursor cell expressed, developmentally down-regulated 9	BX648041	NEDD9	Nucleus	other	NM_006403|NM_182966	Hs.699288///Hs.699288///Hs.699288///Hs.699288///Hs.699288///Hs.699288
122.8	232.1	1.890	127.8	285.7	2.236	2.063	activating transcription factor 3	AB209032	ATF3	Nucleus	transcription regulator	NM_001040619|NM_004024|NM_001674|	Hs.460///Hs.460///Hs.460///Hs.460///Hs.460///Hs.460///Hs.460///Hs.460///Hs.460///Hs.460///Hs.4
27.4	59.8	2.182	27.3	52.9	1.937	2.060	pro-melanin-concentrating hormone	BC018048	PMCH	Extracellular Space	other	NM_002674	Hs.707990///Hs.707990///Hs.707990
221.9	458.4	2.066	310.0	636.0	2.051	2.058							
883.9	2105.5	2.382	1131.9	1953.5	1.726	2.054	ubiquitin-conjugating enzyme E2, J1 (UBC6 homolog, yeast)	AJ420578	UBE2J1	Cytoplasm	enzyme	NM_016021	Hs.163776///Hs.163776///Hs.163776
369.8	639.8	1.730	397.3	932.1	2.346	2.038	nuclear factor of activated T-cells, cytoplasmic, calcineurin-dependent 2	U43341	NFATC2	Nucleus	transcription regulator	NM_012340|NM_173091	Hs.356321///Hs.356321///Hs.356321///Hs.356321///Hs.356321
102.8	236.3	2.299	109.6	194.1	1.771	2.035	ecto-NOX disulfide-thiol exchanger 1	BC024178	ENOX1	Unknown	other	NM_017993	Hs.128258///Hs.128258///Hs.128258///Hs.128258
1017.4	2261.6	2.223	1712.3	3156.3	1.843	2.033	erbb2 interacting protein	AF263744	ERBB2IP	Extracellular Space	cytokine	NM_018695|NM_001006600	Hs.591774///Hs.591774///Hs.591774///Hs.591774///Hs.591774///Hs.591774///Hs.591774///Hs.5
48.4	79.5	1.643	49.5	118.6	2.398	2.020		AK309369		Unknown	other		
822.6	1477.2	1.796	613.1	1376.5	2.245	2.020	HEG homolog 1 (zebrafish)		HEG1	Unknown	other	NM_020733	Hs.477420///Hs.477420
166.4	357.3	2.148	196.8	365.4	1.856	2.002	DNA-damage-inducible transcript 4-like	BC013592	DDIT4L	Unknown	other	NM_145244	Hs.480378///Hs.480378///Hs.480378
515.3	1070.7	2.078	629.5	1181.9	1.877	1.978	myocyte enhancer factor 2A	AL831995	MEF2A	Nucleus	transcription regulator	NM_005587	Hs.709225///Hs.709225///Hs.709225///Hs.709225///Hs.709225
37.4	69.6	1.863	44.1	91.9	2.083	1.973							
71.1	158.1	2.223	78.2	133.0	1.701	1.962		BX640843					
643.3	1070.4	1.664	293.8	663.7	2.259	1.961	synaptojanin 2	AF318616	SYNJ2	Cytoplasm	phosphatase	NM_003898	Hs.434494///Hs.434494///Hs.434494///Hs.434494///Hs.434494
1563.0	3113.2	1.992	1765.4	3390.9	1.921	1.956	radixin	DQ916738	RDX	Cytoplasm	other	NM_002906	Hs.263671///Hs.263671///Hs.263671
201.8	415.2	2.057	84.8	156.2	1.842	1.950	coiled-coil domain containing 69	AL713742	CCDC69	Unknown	other	NM_015621	Hs.655336///Hs.655336///Hs.655336///Hs.655336
256.1	488.6	1.908	169.8	337.4	1.987	1.947	H2.0-like homeobox	BC007294	HLX	Nucleus	transcription regulator	NM_021958	Hs.74870///Hs.645593///Hs.74870///Hs.645593///Hs.74870///Hs.645593///Hs.74870///Hs.64559
295.4	518.0	1.754	397.8	844.7	2.124	1.939		AB011085	KIAA0513	Unknown	other	NM_014732	Hs.301658///Hs.301658///Hs.301658
661.7	1136.8	1.718	783.0	1688.9	2.157	1.937	bone morphogenetic protein 2		BMP2	Extracellular Space	growth factor	NM_001200	Hs.73853///Hs.73853
2360.5	3915.5	1.659	2176.9	4791.3	2.201	1.930	integrin, alpha 2 (CD49B, alpha 2 subunit of VLA-2 receptor)		ITGA2	Plasma Membrane	other	NM_002203	Hs.482077///Hs.482077
321.2	597.0	1.859	448.9	895.0	1.994	1.926	spectrin repeat containing, nuclear envelope 2	AF435011	SYNE2	Nucleus	other	NM_182914|NM_015180|NM_182913|NM_182910	Hs.525392///Hs.525392///Hs.525392///Hs.525392///Hs.525392///Hs.525392///Hs.525392///Hs.525392///Hs.525392///Hs.525392///Hs.525392///Hs.525392
198.8	358.7	1.804	421.7	863.1	2.047	1.925	solute carrier family 9 (sodium/hydrogen exchanger), member 9	BC035779	SLC9A9	Cytoplasm	other	NM_173653	Hs.302257///Hs.302257///Hs.302257
349.4	688.1	1.969	210.2	393.4	1.872	1.920	pleckstrin homology-like domain, family B, member 1	AB094090	PHLDB1	Unknown	other	NM_015157	Hs.504062///Hs.504062///Hs.504062///Hs.504062///Hs.504062///Hs.504062///Hs.504062
128.2	213.9	1.668	152.1	328.1	2.158	1.913	polymerase (DNA directed), gamma 2, accessory subunit	AF142992	POLG2	Cytoplasm	enzyme	NM_007215	Hs.437009///Hs.437009///Hs.437009
216.9	361.8	1.668	261.9	563.2	2.151	1.909	caspase 12 (gene/pseudogene)	AF486844	CASP12	Cytoplasm	other	NR_000035	Hs.476989///Hs.476989///Hs.476989
1736.5	3525.8	2.030	2243.5	3989.0	1.778	1.904	jagged 1 (Alagille syndrome)	AF003837	JAG1	Extracellular Space	growth factor	NM_000214	Hs.224012///Hs.224012///Hs.224012
129.4	226.4	1.750	137.6	281.2	2.043	1.897	replication protein A4, 34kDa	U24186	RPA4	Nucleus	other	NM_013347	Hs.659349///Hs.659349///Hs.659349
115.0	219.1	1.906	269.9	509.0	1.886	1.896	cholesterol 25-hydroxylase	AF059214	CH25H	Cytoplasm	enzyme	NM_003956	Hs.47357///Hs.47357///Hs.47357
4712.8	7826.2	1.661	4047.8	8624.9	2.131	1.896	podocalyxin-like	U97519	PODXL	Plasma Membrane	kinase	NM_001018111|NM_005397	Hs.16426///Hs.16426///Hs.16426///Hs.16426///Hs.16426
2143.6	3832.1	1.788	2546.9	5048.7	1.982	1.885	integrin, alpha V (vitronectin receptor, alpha polypeptide, antigen CD51)	M14648	ITGAV	Nucleus	transcription regulator	NM_002210	Hs.436873///Hs.436873///Hs.436873///Hs.436873
337.2	646.1	1.916	758.2	1405.3	1.853	1.885	AT rich interactive domain 5B (MRF1-like)	BC015120	ARID5B	Plasma Membrane	other	NM_032199	Hs.535297///Hs.535297///Hs.535297///Hs.535297
50.2	80.6	1.605	83.6	180.3	2.157	1.881	complement component 9	BC020721	C9	Extracellular Space	other	NM_001737	Hs.654443///Hs.654443///Hs.654443
86.0	173.2	2.015	92.1	159.4	1.731	1.873		AB027122					
944.8	1678.9	1.777	843.0	1654.7	1.963	1.870	adipose differentiation-related protein	BC005127	ADFP	Plasma Membrane	other	NM_001122	Hs.3416///Hs.3416///Hs.3416///Hs.3416///Hs.3416
286.4	573.5	2.003	187.3	324.1	1.730	1.867	vascular endothelial growth factor C	BC063685	VEGFC	Extracellular Space	growth factor	NM_005429	Hs.435215///Hs.435215///Hs.435215
833.8	1521.6	1.825	711.1	1348.8	1.897	1.861	Rho-related BTB domain containing 3	BC041337	RHOBTB3	Unknown	enzyme	NM_014899	Hs.445030///Hs.445030///Hs.445030
280.4	572.4	2.041	258.9	434.9	1.680	1.861	zinc finger protein 618	BC036039	ZNF618	Unknown	other	NM_133374	Hs.349208///Hs.349208///Hs.349208///Hs.349208///Hs.349208
44.1	72.3	1.639	48.5	100.4	2.073	1.856	RNA, U4 small nuclear 2		RNU4-2	Unknown	other	NR_003137	
627.4	1323.4	2.109	874.8	1400.8	1.601	1.855	ral guanine nucleotide dissociation stimulator-like 1	AF186780	RGL1	Cytoplasm	other	NM_015149	Hs.497148///Hs.497148///Hs.497148///Hs.497148///Hs.497148
413.1	745.8	1.805	465.3	885.8	1.904	1.854	telomerase-associated protein 1	BC126107	TEP1	Nucleus	enzyme	NM_007110	Hs.508835///Hs.508835///Hs.508835///Hs.508835
233.9	424.1	1.813	299.5	554.1	1.850	1.832	solute carrier family 16, member 13 (monocarboxylic acid transporter 13)	BC142618	SLC16A13	Unknown	other	NM_201566	Hs.448010///Hs.448010///Hs.448010
349.2	647.1	1.853	476.7	859.7	1.803	1.828	slingshot homolog 1 (Drosophila)	AB072356	SSH1	Cytoplasm	phosphatase	NM_018984	Hs.199763///Hs.199763///Hs.199763///Hs.199763///Hs.199763///Hs.199763
120.5	212.8	1.767	107.3	202.4	1.887	1.827	chromosome 9 open reading frame 131	AK090398	C9orf131	Unknown	other		
36.7	60.1	1.640	40.0	80.1	2.004	1.822							
402.0	665.1	1.655	1075.6	2139.9	1.990	1.822	chromosome 20 open reading frame 74	DQ310704	C20orf74	Unknown	other	NM_020343	Hs.472285///Hs.472285///Hs.472285
594.1	1013.7	1.706	334.2	644.3	1.928	1.817	zinc finger protein 436	BC056400	ZNF436	Nucleus	other	NM_001077195|NM_030634	Hs.293798///Hs.293798///Hs.293798///Hs.293798///Hs.293798///Hs.293798///Hs.293798
2433.4	4270.5	1.755	2205.5	4132.5	1.874	1.814	v-ets erythroblastosis virus E26 oncogene homolog 1 (avian)	BX640634	ETS1	Nucleus	transcription regulator	NM_005238	Hs.369438///Hs.369438///Hs.369438///Hs.369438///Hs.369438
100.7	196.8	1.953	66.2	110.6	1.670	1.812	vasoactive intestinal peptide	BC009794	VIP	Extracellular Space	other	NM_003381|NM_194435	Hs.53973///Hs.53973///Hs.53973///Hs.53973///Hs.53973
656.7	1063.7	1.620	645.3	1283.3	1.989	1.804	dehydrogenase/reductase (SDR family) member 3	BC002730	DHRS3	Cytoplasm	enzyme	NM_004753	Hs.289347///Hs.289347///Hs.289347
334.7	666.4	1.991	520.7	841.4	1.616	1.803	cytochrome P450, family 1, subfamily A, polypeptide 1	BC023019	CYP1A1	Cytoplasm	enzyme	NM_000499	Hs.72912///Hs.72912///Hs.72912///Hs.72912///Hs.72912///Hs.72912
276.5	514.5	1.861	305.8	532.8	1.742	1.802	dual specificity phosphatase 16	AF506796	DUSP16	Nucleus	phosphatase	NM_030640	Hs.536535///Hs.536535///Hs.536535///Hs.536535
210.2	404.5	1.924	211.9	354.4	1.673	1.798	zinc finger protein 618	AF495724	ZNF618	Unknown	other	NM_133374	Hs.349208///Hs.349208
460.2	823.7	1.790	341.6	615.9	1.803	1.796	protein tyrosine phosphatase type IVA, member 3 | similar to protein tyrosine phosphatase type IVA, member 3	AF041434	PTP4A3|LOC100131062	Plasma Membrane	phosphatase	NM_032611|NM_007079	Hs.43666///Hs.43666///Hs.43666///Hs.43666///Hs.43666///Hs.43666
92.9	170.2	1.832	125.2	219.0	1.750	1.791	G protein-coupled receptor 137C		GPR137C	Unknown	other	NM_001099652	Hs.416214///Hs.416214
398.7	646.3	1.621	377.3	738.3	1.957	1.789	leucine rich repeat containing 8 family, member C	BC036122	LRRC8C	Cytoplasm	other	NM_032270	Hs.657493///Hs.657493///Hs.657493
206.0	376.4	1.827	116.1	202.3	1.742	1.785	integrin, alpha 10	AF112345	ITGA10	Plasma Membrane	other	NM_003637	Hs.158237///Hs.158237///Hs.158237
812.2	1435.2	1.767	678.9	1223.3	1.802	1.785	myosin IE	BC098392	MYO1E	Cytoplasm	enzyme	NM_004998	Hs.654506///Hs.654506///Hs.654506
342.0	670.8	1.961	730.7	1174.0	1.607	1.784	phosphatidic acid phosphatase type 2A	AF014402	PPAP2A	Plasma Membrane	phosphatase	NM_003711|NM_176895	Hs.696231///Hs.696231///Hs.696231///Hs.696231///Hs.696231///Hs.696231///Hs.696231
1697.0	2716.1	1.600	333.8	655.5	1.964	1.782	NAD(P)H dehydrogenase, quinone 1	J03934	NQO1	Cytoplasm	enzyme	NM_000903|NM_001025433|NM_0010254	Hs.406515///Hs.406515///Hs.406515///Hs.406515///Hs.406515///Hs.406515///Hs.406515
660.3	1087.1	1.646	539.2	1018.7	1.889	1.768	ADAM metallopeptidase with thrombospondin type 1 motif, 9	BC130578	ADAMTS9	Extracellular Space	peptidase	NM_182920	Hs.656071///Hs.656071///Hs.656071
875.8	1501.1	1.714	489.4	888.8	1.816	1.765	transmembrane protein 173	AK095896	TMEM173	Unknown	other	NM_198282	Hs.379754///Hs.379754///Hs.379754
376.7	625.9	1.661	311.0	577.0	1.855	1.758	interleukin 4 receptor		IL4R	Plasma Membrane	transmembrane receptor	NM_000418	Hs.513457///Hs.513457///Hs.513457///Hs.513457
81.8	152.9	1.869	75.8	122.9	1.621	1.745	potassium inwardly-rectifying channel, subfamily J, member 2	AF153820	KCNJ2	Plasma Membrane	ion channel	NM_000891	Hs.1547///Hs.1547///Hs.1547
1927.4	3332.7	1.729	1618.9	2832.3	1.750	1.739	sequestosome 1	BC001874	SQSTM1	Cytoplasm	transcription regulator	NM_003900	Hs.437277///Hs.437277///Hs.437277
264.1	483.8	1.832	311.6	511.3	1.641	1.737	arginase, type II	BC029050	ARG2	Cytoplasm	enzyme	NM_001172	Hs.708024///Hs.708024///Hs.708024
51.4	86.5	1.685	66.0	117.3	1.776	1.731							
721.0	1206.5	1.673	928.5	1656.4	1.784	1.729	solute carrier family 4, sodium bicarbonate cotransporter, member 7	AF047033	SLC4A7	Plasma Membrane	transporter	NM_003615	Hs.250072///Hs.250072///Hs.250072///Hs.250072///Hs.250072
191.8	314.9	1.642	247.9	448.0	1.808	1.725	Rho GTPase activating protein 25	AK290396	ARHGAP25	Unknown	other	NM_014882|NM_001007231	Hs.531807///Hs.531807///Hs.531807///Hs.531807
169.0	300.4	1.777	187.6	311.9	1.662	1.720	suppression of tumorigenicity 5	BC036655	ST5	Unknown	enzyme	NM_005418|NM_213618|NM_139157	Hs.117715///Hs.117715///Hs.117715///Hs.117715///Hs.117715///Hs.117715///Hs.117715///Hs.1
116.9	208.7	1.785	160.0	263.8	1.649	1.717	transforming growth factor, beta 3	J03241	TGFB3	Extracellular Space	growth factor	NM_003239	Hs.592317///Hs.592317///Hs.592317
655.5	1085.2	1.655	2786.2	4886.3	1.754	1.705		M98398	CD36	Plasma Membrane	transmembrane receptor	NM_001001548|NM_001001547|NM_0000	Hs.120949///Hs.120949///Hs.120949///Hs.120949///Hs.120949///Hs.120949///Hs.120949
295.4	530.1	1.794	337.8	543.3	1.608	1.701	transmembrane channel-like 7	BC036205	TMC7	Unknown	other	NM_024847	Hs.187377///Hs.187377///Hs.187377
136.6	226.6	1.659	149.2	259.4	1.739	1.699	family with sequence similarity 122C	BC017868|BC063491|BC065225|AK130840|BC016784|AK090	FAM122C	Unknown	other		Hs.269127///Hs.269127///Hs.269127///Hs.269127///Hs.269127///Hs.269127///Hs.269127///Hs.269127
577.0	949.9	1.646	408.4	712.4	1.744	1.695	protocadherin 12	AF240635	PCDH12	Plasma Membrane	other	NM_016580	Hs.439474///Hs.439474///Hs.439474
198.0	322.5	1.629	268.1	470.4	1.755	1.692	transmembrane and tetratricopeptide repeat containing 2	BC093852	TMTC2	Cytoplasm	other	NM_152588	Hs.577775///Hs.577775///Hs.577775
232.9	387.5	1.664	833.5	1380.0	1.656	1.660	Ras protein-specific guanine nucleotide-releasing factor 2	BC126112	RASGRF2	Cytoplasm	other	NM_006909	Hs.162129///Hs.162129///Hs.162129

825.3	1345.4	1.630	1112.8	1856.7	1.668	1.649	ADAM metallopeptidase domain 17 (tumor necrosis factor, alpha, converting enzyme)	U92649	ADAM17	Plasma Membrane	peptidase	NM_003183	Hs.404914///Hs.404914///Hs.404914
276.3	463.2	1.676	200.9	325.6	1.621	1.648	ADP-ribosylation factor-like 4C	BC089043	ARL4C	Nucleus	enzyme	NM_005737	Hs.699342///Hs.709513///Hs.699342///Hs.709513///Hs.699342///Hs.709513///Hs.699342///Hs.7
585.0	977.6	1.671	637.6	1030.7	1.616	1.644	diaphanous homolog 2 (Drosophila)	Y15909	DIAPH2	Cytoplasm	other	NM_006729|NM_007309	Hs.226483///Hs.226483///Hs.226483///Hs.226483///Hs.226483///Hs.226483///Hs.226483///Hs.2
40.7	70.6	1.734	111.9	68.5	0.612	1.173							
45.8	78.7	1.717	87.7	51.3	0.585	1.151							
24.0	44.5	1.856	55.2	23.4	0.423	1.140							
453.9	754.1	1.661	1077.7	661.1	0.613	1.137							
30.0	50.9	1.696	54.2	28.5	0.527	1.111							
75.9	124.9	1.645	115.9	64.2	0.554	1.099							
69.9	113.2	1.621	79.0	41.6	0.527	1.074							
543.5	337.4	0.621	466.1	287.5	0.617	0.619	ectodermal-neural cortex (with BTB-like domain)	AK096578	ENC1	Nucleus	peptidase	NM_003633	Hs.104925///Hs.104925///Hs.104925
1405.7	851.8	0.606	2618.0	1603.9	0.613	0.609	interferon responsive gene 15	BC098348	IFRG15	Unknown	other	NM_022347	Hs.655655///Hs.655655
157.4	95.9	0.610	1045.2	626.0	0.599	0.604	polycystic kidney and hepatic disease 1 (autosomal recessive)-like 1	AY219181	PKHD1L1	Extracellular Space	other	NM_177531	Hs.170128///Hs.170128///Hs.170128
366.5	221.0	0.603	624.8	376.2	0.602	0.603	phosphatidylinositol glycan anchor biosynthesis, class X	AK000529	PIGX	Unknown	other	NM_017861	Hs.223296///Hs.223296///Hs.223296///Hs.223296///Hs.223296
437.4	253.7	0.580	488.8	305.0	0.624	0.602	RIO kinase 2 (yeast)	BC000953	RIOK2	Unknown	kinase	NM_018343	Hs.27021///Hs.27021///Hs.27021
180.7	111.5	0.617	455.9	267.6	0.587	0.602	ADAM metallopeptidase with thrombospondin type 1 motif, 18	BC063283	ADAMTS18	Extracellular Space	peptidase	NM_199355	Hs.188746///Hs.188746///Hs.188746///Hs.188746
856.3	515.3	0.602	838.0	498.4	0.595	0.598	cAMP responsive element binding protein 3-like 2	BC110813	CREB3L2			NM_194071	Hs.490273///Hs.490273///Hs.490273///Hs.490273
928.7	557.5	0.600	1306.8	756.3	0.579	0.590	Rho-guanine nucleotide exchange factor	AK025816	RGNEF	Unknown	other	NM_001080479	Hs.482521///Hs.482521///Hs.482521
200.3	117.9	0.589	460.6	269.7	0.586	0.587	EPH receptor B1	L40636	EPHB1	Plasma Membrane	kinase	NM_004441	Hs.116092///Hs.116092///Hs.116092///Hs.116092
96.7	54.5	0.564	107.4	65.5	0.610	0.587	ankyrin repeat domain 20B | coiled-coil domain containing 29 | hypothetical LOC644249		ANKRD20B|CCDC29|RP11-195B21.3			NR_003366|NM_001098806|XR_041271|XR_037208|XR_037226|XR_037144|XM_00	Hs.699146///Hs.699146///Hs.699146///Hs.699146///Hs.699146///Hs.699146///Hs.699146
468.2	282.9	0.604	578.2	328.4	0.568	0.586	KDEL (Lys-Asp-Glu-Leu) endoplasmic reticulum protein retention receptor 3	BC001277	KDELR3	Cytoplasm	transporter	NM_006855|NM_016657	Hs.709898///Hs.709898///Hs.709898///Hs.709898
387.1	212.1	0.548	381.8	236.2	0.619	0.583	adenylate kinase 3-like 1 | adenylate kinase 3-like 2	CR456830	AK3L1|AK3L2	Cytoplasm	kinase	NM_001005353|NM_013410|NM_203464	Hs.10862///Hs.592601///Hs.10862///Hs.592601///Hs.10862///Hs.592601///Hs.10862///Hs.592601///Hs.10862///Hs.592601///Hs.10862///Hs.592601///Hs.10862///Hs.592601
474.8	258.7	0.545	938.7	574.6	0.612	0.578	midline 2	Y18880	MID2	Cytoplasm	other	NM_012216|NM_052817	Hs.12256///Hs.12256///Hs.12256///Hs.12256///Hs.12256
207.8	112.0	0.539	531.6	323.2	0.608	0.573	RAB11 family interacting protein 1 (class I)	AY280968	RAB11FIP1	Cytoplasm	other	NM_001002814|NM_001002233|NM_0251	Hs.696035///Hs.696035///Hs.696035///Hs.696035///Hs.696035///Hs.696035///Hs.696035
1166.8	706.8	0.606	1759.9	945.2	0.537	0.571	WW domain containing E3 ubiquitin protein ligase 1	BC036065	WWP1	Cytoplasm	enzyme	NM_007013	Hs.655189///Hs.655189///Hs.655189///Hs.655189///Hs.655189
626.9	327.9	0.523	615.6	372.8	0.606	0.564	CD163 molecule-like 1	AF264014	CD163L1	Plasma Membrane	transmembrane receptor	NM_174941	Hs.631727///Hs.631727///Hs.631727///Hs.631727
2391.9	1311.9	0.548	3449.6	1996.0	0.579	0.564	fibronectin type III domain containing 3B	AB098597	FNDC3B	Unknown	other	NM_022763	Hs.159430///Hs.159430///Hs.159430
2717.4	1679.8	0.618	1625.7	812.9	0.500	0.559	integrin, beta 3 (platelet glycoprotein IIIa, antigen CD61)	U95204	ITGB3	Plasma Membrane	transmembrane receptor	NM_000212	Hs.218040///Hs.218040///Hs.218040
76.9	43.9	0.571	65.1	35.6	0.546	0.558							
1035.9	618.2	0.597	1074.3	558.7	0.520	0.558	phosphodiesterase 2A, cGMP-stimulated	BC040974	PDE2A	Cytoplasm	enzyme	NM_002599	Hs.503163///Hs.503163///Hs.503163///Hs.503163
867.1	476.8	0.550	1669.0	938.0	0.562	0.556	GTPase, IMAP family member 7	BC027613	GIMAP7	Unknown	other	NM_153236	Hs.647074///Hs.647074///Hs.647074
677.5	339.7	0.501	742.8	452.1	0.609	0.555	methylenetetrahydrofolate dehydrogenase (NADP+ dependent) 2, methenyltetrahydrofolate cyclohydrolase	BC015062	MTHFD2	Cytoplasm	enzyme	NM_001040409|NM_006636	Hs.469030///Hs.469030///Hs.469030///Hs.469030///Hs.469030///Hs.469030///Hs.469030
277.9	171.3	0.616	726.9	357.3	0.492	0.554	oxidative stress induced growth inhibitor family member 2	AF061326	OSGIN2	Unknown	other	NM_004337|NM_001126111	Hs.436445///Hs.436445///Hs.436445///Hs.436445
66.2	40.6	0.614	310.2	153.1	0.494	0.554							
269.2	158.8	0.590	410.5	210.8	0.513	0.552	retinitis pigmentosa GTPase regulator	AK291832	RPGR	Cytoplasm	other	NM_000328|NM_001034853	Hs.61438///Hs.61438///Hs.61438///Hs.61438///Hs.61438///Hs.61438///Hs.61438///Hs.61438///Hs
750.0	424.4	0.566	946.3	507.2	0.536	0.551	phosphoglucomutase 1	BC019920	PGM1	Cytoplasm	enzyme	NM_002633	Hs.1869///Hs.1869///Hs.1869///Hs.1869///Hs.1869
3056.8	1612.5	0.528	3661.3	2090.0	0.571	0.549	potassium channel tetramerisation domain containing 12	AF359381	KCTD12	Unknown	ion channel	NM_138444	Hs.644125///Hs.644125///Hs.644125///Hs.644125
437.8	251.8	0.575	796.1	415.1	0.521	0.548	MAP7 domain containing 3	AL832120	MAP7D3	Unknown	other	NM_024597	Hs.446275///Hs.446275///Hs.446275///Hs.446275///Hs.446275///Hs.446275
1610.0	865.4	0.538	2675.3	1488.5	0.556	0.547	pellino homolog 1 (Drosophila)	AF302505	PELI1	Cytoplasm	other	NM_020651	Hs.7886///Hs.7886///Hs.7886///Hs.7886
651.6	349.2	0.536	1919.6	1071.0	0.558	0.547	tetraspanin 12	BC031265	TSPAN12	Unknown	other	NM_012338	Hs.16529///Hs.16529///Hs.16529
178.8	110.5	0.618	955.1	448.6	0.470	0.544	follistatin	BC004107	FST	Extracellular Space	other	NM_006350|NM_013409	Hs.9914///Hs.9914///Hs.9914///Hs.9914///Hs.9914
151.6	90.6	0.598	419.9	204.8	0.488	0.543	interleukin-1 receptor-associated kinase 3	BC057800	IRAK3	Extracellular Space	kinase	NM_007199	Hs.369265///Hs.369265///Hs.369265
1270.5	586.4	0.462	605.2	372.8	0.616	0.539	histone cluster 1, H2bm	BC066244	HIST1H2BM	Nucleus	other	NM_003521	Hs.182432///Hs.182432///Hs.182432
208.6	111.3	0.534	833.0	452.0	0.543	0.538	filamin A interacting protein 1	BX647178	FILIP1	Cytoplasm	other	NM_015687	Hs.696158///Hs.696158///Hs.696158///Hs.696158///Hs.696158
1158.4	572.7	0.494	1679.6	976.7	0.582	0.538	acyl-CoA synthetase long-chain family member 3	BC041692	ACSL3	Cytoplasm	enzyme	NM_004457|NM_203372	Hs.655772///Hs.655772///Hs.655772///Hs.655772///Hs.655772///Hs.655772
1457.7	703.4	0.483	2291.2	1349.0	0.589	0.536	dimethylarginine dimethylaminohydrolase 1	BC033680	DDAH1	Cytoplasm	enzyme	NM_012137	Hs.379858///Hs.379858///Hs.379858
597.3	306.4	0.513	967.4	539.6	0.558	0.535	kalirin, RhoGEF kinase	U94190	KALRN	Cytoplasm	kinase	NM_001024660|NM_003947|NM_007064	Hs.8004///Hs.8004///Hs.8004///Hs.8004///Hs.8004///Hs.8004///Hs.8004///Hs.8004///Hs.8004///Hs
200.4	107.7	0.538	585.0	307.9	0.526	0.532	par-6 partitioning defective 6 homolog gamma (C. elegans)	BC060797	PARD6G	Plasma Membrane	other	NM_032510	Hs.654920///Hs.654920///Hs.654920
325.1	182.7	0.562	853.6	428.0	0.501	0.532	platelet derived growth factor C	AF091434	PDGFC	Extracellular Space	growth factor	NM_016205	Hs.570855///Hs.570855///Hs.570855
723.3	352.4	0.487	1058.1	608.3	0.575	0.531	ubiquitin specific peptidase 13 (isopeptidase T-3)	BC016146	USP13	Unknown	peptidase	NM_003940	Hs.591319///Hs.591319///Hs.591319
139.8	83.8	0.599	383.8	175.6	0.457	0.528	microfibrillar-associated protein 3-like	AK290041	MFAP3L	Unknown	other	NM_021647|NM_001009554	Hs.593942///Hs.593942///Hs.593942///Hs.593942///Hs.593942///Hs.593942
125.9	77.7	0.617	322.9	141.8	0.439	0.528	bone morphogenetic protein 4	BC020546	BMP4	Extracellular Space	growth factor	NM_001202|NM_130851|NM_130850	Hs.68879///Hs.68879///Hs.68879///Hs.68879///Hs.68879///Hs.68879///Hs.68879
86.0	42.9	0.499	49.9	27.4	0.549	0.524							
6383.6	3156.0	0.494	2051.2	1135.6	0.554	0.524	metallothionein 2A	BC007034	MT2A	Cytoplasm	other	NM_005953	Hs.647371///Hs.647371///Hs.647371
297.2	166.7	0.561	408.7	198.9	0.487	0.524	solute carrier family 1 (glutamate/neutral amino acid transporter), member 4	BC026216	SLC1A4	Plasma Membrane	transporter	NM_003038	Hs.654352///Hs.654352///Hs.654352
1389.7	635.2	0.457	1941.4	1134.9	0.585	0.521	GTPase, IMAP family member 8	BC107037	GIMAP8	Unknown	other	NM_175571	Hs.647121///Hs.647121///Hs.647121
352.8	177.8	0.504	372.9	199.4	0.535	0.519	small nucleolar RNA, C/D box 45B | Rab geranylgeranyltransferase, beta subunit	AK128861	SNORD45B|RABGGTB			NR_002748	
373.2	221.1	0.592	446.8	194.9	0.436	0.514	chemokine (C-C motif) ligand 14 | chemokine (C-C motif) ligand 15	BC045165|U58914	CCL14|CCL15			NM_032962|NM_004167|NM_004166|NM_032964|NM_032963|NM_032965	Hs.272493///Hs.656286///Hs.272493///Hs.656286///Hs.272493///Hs.656286///Hs.656286///Hs.656286///Hs.272493///Hs.272493///Hs.656286///Hs.272493///Hs.656286
3789.3	2045.0	0.540	6173.3	3015.3	0.488	0.514	DnaJ (Hsp40) homolog, subfamily B, member 4	BC034721	DNAJB4	Nucleus	other	NM_007034	Hs.13852///Hs.13852///Hs.13852
1457.7	594.6	0.408	1755.4	1086.9	0.619	0.514	serum deprivation response (phosphatidylserine binding protein)	BC016475	SDPR	Plasma Membrane	other	NM_004657	Hs.26530///Hs.26530///Hs.26530
3489.1	1652.1	0.474	5031.9	2784.8	0.553	0.513	netrin 4	AF278532	NTN4	Extracellular Space	other	NM_021229	Hs.201034///Hs.201034///Hs.201034///Hs.201034
714.4	335.1	0.469	1384.6	771.2	0.557	0.513	PRKC, apoptosis, WT1, regulator	BC007018	PAWR	Nucleus	transcription regulator	NM_002583	Hs.643130///Hs.643130///Hs.643130
138.0	64.8	0.470	161.7	87.5	0.541	0.505	transmembrane protein 155	BC047657	TMEM155	Unknown	other	NM_152399	Hs.27524///Hs.27524///Hs.27524///Hs.27524///Hs.27524
245.8	149.6	0.609	752.8	294.9	0.392	0.500	Mov10l1, Moloney leukemia virus 10-like 1, homolog (mouse)	AF285604	MOV10L1	Nucleus	enzyme	NM_018995	Hs.62880///Hs.62880///Hs.62880///Hs.62880///Hs.62880///Hs.62880///Hs.62880///Hs.62880
558.2	314.4	0.563	880.8	382.0	0.434	0.498	WAS protein family, member 3	BC050283	WASF3	Cytoplasm	other	NM_006646	Hs.635221///Hs.635221///Hs.635221///Hs.635221///Hs.635221
109.3	59.6	0.545	261.2	117.3	0.449	0.497	phospholipase C, epsilon 1	BC151854	PLCE1	Cytoplasm	enzyme	NM_016341	Hs.655033///Hs.655033///Hs.655033///Hs.655033///Hs.655033///Hs.655033
740.3	307.5	0.415	567.7	327.7	0.577	0.496		D84276	CD38	Plasma Membrane	enzyme	NM_001775	Hs.479214///Hs.479214///Hs.479214
715.4	440.3	0.615	1596.4	580.0	0.363	0.489	microRNA 21	AY699265	MIRN21	Unknown	microRNA		
1677.0	787.6	0.470	2188.8	1106.2	0.505	0.488	mannosyl (alpha-1,6-)-glycoprotein beta-1,6-N-acetyl-glucosaminyltransferase	D17716	MGAT5	Cytoplasm	enzyme	NM_002410	Hs.651869///Hs.651869///Hs.651869
166.8	102.3	0.613	305.3	109.6	0.359	0.486						NC_001807	
3718.5	1726.3	0.464	4726.7	2364.0	0.500	0.482	cyclin D2	D13639	CCND2	Nucleus	other	NM_001759	Hs.376071///Hs.376071///Hs.376071
591.5	346.6	0.586	1977.0	723.2	0.366	0.476	small nuclear RNA activating complex, polypeptide 1, 43kDa	BC019038	SNAPC1	Nucleus	other	NM_003082	Hs.179312///Hs.179312///Hs.179312
2773.7	1239.8	0.447	4226.8	2118.3	0.501	0.474	nuclear factor I/B	BX648845	NFIB	Nucleus	transcription regulator	NM_005596	Hs.709255///Hs.709255///Hs.709255///Hs.709255///Hs.709255///Hs.709255///Hs.709255///Hs.709255///Hs.709255///Hs.709255///Hs.709255///Hs.709255
218.0	112.9	0.518	244.5	103.5	0.423	0.471		BC125017|AB051559|AK0237	KIAA1772	Unknown	other		Hs.149020///Hs.149020///Hs.149020///Hs.149020///Hs.149020
129.3	70.0	0.541	245.3	98.0	0.399	0.470	arrestin domain containing 4	BC028704	ARRDC4	Unknown	other	NM_183376	Hs.6093///Hs.6093///Hs.6093
269.7	156.3	0.579	852.7	303.6	0.356	0.468		AK001150					
154.5	70.5	0.456	206.9	98.7	0.477	0.467	small nucleolar RNA, C/D box 114-2		SNORD114-2	Unknown	other	NR_003194	
556.9	281.9	0.506	1083.7	453.5	0.418	0.462	tribbles homolog 2 (Drosophila)	AY245544	TRIB2	Plasma Membrane	kinase	NM_021643	Hs.467751///Hs.467751///Hs.467751///Hs.467751
669.5	226.8	0.339	574.4	335.4	0.584	0.461	tolloid-like 1	U91963	TLL1	Extracellular Space	peptidase	NM_012464	Hs.106513///Hs.106513///Hs.106513
9276.2	4093.9	0.441	11489.0	5526.3	0.481	0.461	aldehyde dehydrogenase 1 family, member A1	BC001505	ALDH1A1	Cytoplasm	enzyme	NM_000689	Hs.76392///Hs.76392///Hs.76392///Hs.76392
459.4	216.2	0.470	1230.5	533.3	0.433	0.452	v-maf musculoaponeurotic fibrosarcoma oncogene homolog (avian)	BC081542	MAF	Nucleus	transcription regulator	NM_001031804|NM_005360	Hs.709603///Hs.709603///Hs.709603///Hs.709603///Hs.709603
136.5	72.2	0.529	302.1	113.1	0.374	0.452	glycine receptor, beta	BC032635	GLRB	Plasma Membrane	ion channel	NM_000824	Hs.32973///Hs.32973///Hs.32973
1908.0	575.0	0.301	253.5	152.2	0.601	0.451	myelin protein zero-like 2	BC017774	MPZL2	Plasma Membrane	other	NM_144765|NM_005797	Hs.116651///Hs.116651///Hs.116651///Hs.116651
210.3	71.3	0.339	1069.4	596.1	0.557	0.448		X77690					
616.1	262.1	0.425	1557.0	716.1	0.460	0.443	UDP-glucose ceramide glucosyltransferase	BC038711	UGCG	Cytoplasm	enzyme	NM_003358	Hs.304249///Hs.593014///Hs.304249///Hs.593014///Hs.304249///Hs.593014
1677.0	875.6	0.522	1585.0	572.3	0.361	0.442		S77921				NC_001807	
238.5	133.1	0.558	1044.0	336.0	0.322	0.440	UDP-N-acetyl-alpha-D-galactosamine:polypeptide N-acetylgalactosaminyltransferase-like 4	BC037341	GALNTL4	Cytoplasm	enzyme	NM_198516	Hs.655152///Hs.655152///Hs.655152
614.3	304.5	0.496	1318.8	499.5	0.379	0.437	nuclear receptor subfamily 2, group F, member 1	BC017493	NR2F1	Nucleus	ligand-dependent nuclear receptor	NM_005654	Hs.519445///Hs.519445///Hs.519445

91.3	41.2	0.451	750.7	313.2	0.417	0.434	ADP-ribosyltransferase 4 (Dombrock blood group)	AK291662	ART4	Nucleus	enzyme	NM_021071	Hs.591158///Hs.655792///Hs.668803///Hs.591158///Hs.655792///Hs.668803///Hs.591158///Hs.6
411.0	160.4	0.390	1281.2	597.3	0.466	0.428	multiple C2 domains, transmembrane 1	AY656715	MCTP1	Unknown	other	NM_024717|NM_001002796	Hs.655087///Hs.655087///Hs.655087///Hs.655087///Hs.655087///Hs.655087
70.8	30.6	0.432	132.8	56.2	0.423	0.428	transmembrane protein 100	BC010128	TMEM100	Unknown	other	NM_001099640|NM_018286	Hs.173233///Hs.173233///Hs.173233///Hs.173233
1104.4	387.6	0.351	1912.0	963.6	0.504	0.427	thrombospondin, type I, domain containing 7A	BC016980	THSD7A	Unknown	other	NM_015204	Hs.120855///Hs.648482///Hs.120855///Hs.648482///Hs.120855///Hs.648482
1213.4	370.5	0.305	3201.8	1694.4	0.529	0.417	ephrin-A5	U26403	EFNA5	Plasma Membrane	kinase	NM_001962	Hs.658451///Hs.658451///Hs.658451
1408.8	672.9	0.478	2500.2	882.3	0.353	0.415	3'-phosphoadenosine 5'-phosphosulfate synthase 2	AF074331	PAPSS2	Cytoplasm	enzyme	NM_004670|NM_001015880	Hs.524491///Hs.524491///Hs.524491///Hs.524491///Hs.524491
118.3	45.0	0.380	286.7	125.5	0.438	0.409	sorbin and SH3 domain containing 2	AF090937	SORBS2	Nucleus	other		
250.0	118.7	0.475	355.1	121.6	0.343	0.409	growth hormone receptor	X06562	GHR	Plasma Membrane	transmembrane receptor	NM_000163	Hs.125180///Hs.684631///Hs.125180///Hs.684631///Hs.125180///Hs.684631///Hs.125180///Hs.6
316.6	116.9	0.369	363.0	157.9	0.435	0.402	solute carrier family 45, member 4	BC033223	SLC45A4	Unknown	other		Hs.372492///Hs.372492
339.7	166.8	0.491	702.3	219.4	0.312	0.402	phospholipid scramblase 1	AB006746	PLSCR1	Plasma Membrane	enzyme	NM_021105	Hs.130759///Hs.130759///Hs.130759
1533.1	533.2	0.348	7197.9	3220.8	0.447	0.398	hedgehog interacting protein	AY009951	HHIP	Plasma Membrane	other	NM_022475	Hs.507991///Hs.507991///Hs.507991
574.4	230.5	0.401	718.6	272.5	0.379	0.390	protein phosphatase 1, regulatory (inhibitor) subunit 3B	BC043388	PPP1R3B	Unknown	other	NM_024607	Hs.458513///Hs.458513///Hs.458513
1060.2	426.8	0.403	2297.0	865.7	0.377	0.390	tumor necrosis factor receptor superfamily, member 21	BC021572	TNFRSF21	Plasma Membrane	other	NM_014452	Hs.443577///Hs.443577///Hs.443577
265.0	129.6	0.489	650.1	175.7	0.270	0.380	myosin light chain kinase 2	BC092413	MYLK2	Cytoplasm	kinase	NM_033118	Hs.86092///Hs.86092///Hs.86092///Hs.86092
942.7	379.7	0.403	1658.4	587.1	0.354	0.378	selenoprotein P, plasma, 1	BC015875	SEPP1	Extracellular Space	other	NM_005410|NM_001085486|NM_0010937	Hs.275775///Hs.709405///Hs.275775///Hs.709405///Hs.275775///Hs.709405///Hs.275775///Hs.7
144.5	65.9	0.456	1028.1	302.6	0.294	0.375	solute carrier family 2 (facilitated glucose transporter), member 12	AL833602	SLC2A12	Unknown	transporter	NM_145176	Hs.486508///Hs.486508///Hs.486508
984.6	405.4	0.412	2660.8	889.5	0.334	0.373	exocyst complex component 6	AK128190	EXOC6	Unknown	transporter	NM_019053|NM_001013848	Hs.655657///Hs.655657///Hs.655657///Hs.655657///Hs.655657///Hs.655657///Hs.655657
987.9	201.6	0.204	2351.0	1271.3	0.541	0.372	coxsackie virus and adenovirus receptor	BC010536	CXADR	Plasma Membrane	transmembrane receptor	NM_001338	Hs.705503///Hs.705503///Hs.705503///Hs.705503///Hs.705503///Hs.705503///Hs.705503
175.2	84.5	0.483	520.5	135.9	0.261	0.372	collagen, type VIII, alpha 1	AF170702	COL8A1	Extracellular Space	other	NM_001850|NM_020351	Hs.654548///Hs.654548///Hs.654548///Hs.654548///Hs.654548
767.2	262.8	0.343	2075.7	795.9	0.383	0.363	phosphoglucomutase 5 pseudogene 2	BC033073	PGM5P2			NR_002836	
148.8	70.0	0.471	294.0	73.2	0.249	0.360		AK091822	FLJ34503	Unknown	other		Hs.712110///Hs.712110
667.1	340.5	0.510	1461.3	284.8	0.195	0.353	Ras association (RalGDS/AF-6) domain family member 2	AY154470	RASSF2	Nucleus	other	NM_014737|NM_170774	Hs.631504///Hs.631504///Hs.631504///Hs.631504///Hs.631504///Hs.631504
841.1	276.7	0.329	2322.6	873.0	0.376	0.352	phosphoglucomutase 5 pseudogene 2	BC033073	PGM5P2			NR_002836	
248.5	85.9	0.346	488.0	157.5	0.323	0.334	interleukin 1 receptor, type I	M27492	IL1R1	Plasma Membrane	transmembrane receptor	NM_000877	Hs.701982///Hs.701982///Hs.701982
224.0	78.5	0.351	1203.3	379.2	0.315	0.333	monoamine oxidase A	BC044787	MAOA	Cytoplasm	enzyme	NM_000240	Hs.183109///Hs.183109///Hs.183109
55.0	28.8	0.524	280.1	38.7	0.138	0.331	UDP-Gal:betaGlcNAc beta 1,3-galactosyltransferase, polypeptide 2	BC022507	B3GALT2	Cytoplasm	enzyme	NM_003783	Hs.518834///Hs.518834///Hs.518834
183.5	60.4	0.329	643.8	205.8	0.320	0.324	phosphoglucomutase 5		PGM5	Cytoplasm	enzyme	NM_021965	Hs.307835///Hs.307835///Hs.307835///Hs.307835
535.6	163.2	0.305	934.4	295.6	0.316	0.311	transient receptor potential cation channel, subfamily C, member 6	AF080394	TRPC6	Plasma Membrane	ion channel	NM_004621	Hs.159003///Hs.159003///Hs.159003///Hs.159003///Hs.159003
2859.9	520.9	0.182	6140.0	2600.0	0.423	0.303	phospholipid scramblase 4	AF199023	PLSCR4	Plasma Membrane	enzyme	NM_020353	Hs.477869///Hs.477869///Hs.477869///Hs.477869
746.5	198.5	0.266	1838.1	619.9	0.337	0.302	DEP domain containing 2	AJ437636	DEPDC2	Unknown	other	NM_024870|NM_025170	Hs.591867///Hs.591867///Hs.591867///Hs.591867///Hs.591867///Hs.591867///Hs.591867
453.6	154.5	0.340	1334.0	342.8	0.257	0.299	leucine rich repeat containing 1	AK021896	LRRC1	Cytoplasm	other	NM_018214	Hs.700747///Hs.700747///Hs.700747///Hs.700747///Hs.700747
4575.5	1195.3	0.261	4580.7	1523.7	0.333	0.297	connective tissue growth factor	BC087839	CTGF	Extracellular Space	growth factor	NM_001901	Hs.591346///Hs.591346///Hs.591346
2556.2	488.1	0.191	3299.8	1255.2	0.380	0.286	cysteine-rich, angiogenic inducer, 61	BC009199	CYR61	Extracellular Space	other	NM_001554	Hs.8867///Hs.8867///Hs.8867///Hs.8867
1891.5	283.4	0.150	2467.3	1036.5	0.420	0.285	pentraxin-related gene, rapidly induced by IL-1 beta	BC039733	PTX3	Extracellular Space	other	NM_002852	Hs.591286///Hs.591286///Hs.591286
264.8	84.1	0.317	919.9	207.0	0.225	0.271	Kv channel interacting protein 1	AY780424	KCNIP1	Plasma Membrane	ion channel	NM_001034837|NM_014592|NM_0010348 38	Hs.484111///Hs.689588///Hs.484111///Hs.689588///Hs.484111///Hs.689588///Hs.484111///Hs.6 89588///Hs.484111///Hs.689588///Hs.484111///Hs.689588///Hs.484111///Hs.689588
1051.8	194.5	0.185	2047.1	715.6	0.350	0.267	sorbin and SH3 domain containing 2	AK056628	SORBS2	Nucleus	other	NM_021069|NM_003603	Hs.655143///Hs.655143///Hs.655143///Hs.655143///Hs.655143///Hs.655143///Hs.655143///Hs.6
3166.0	569.9	0.180	7735.7	2604.1	0.337	0.258	ankyrin repeat domain 1 (cardiac muscle)	BC018667	ANKRD1	Cytoplasm	transcription regulator	NM_014391	Hs.448589///Hs.448589///Hs.448589
837.7	183.8	0.219	881.0	256.4	0.291	0.255	solute carrier family 45, member 4	AB032952	SLC45A4	Unknown	other	NM_001080431	Hs.372492///Hs.372492///Hs.372492
307.6	61.2	0.199	418.6	120.0	0.287	0.243	phosphodiesterase 1A, calmodulin-dependent	BC022480	PDE1A	Cytoplasm	enzyme	NM_005019|NM_001003683	Hs.191046///Hs.680373///Hs.191046///Hs.680373///Hs.191046///Hs.680373///Hs.191046///Hs.6 80373///Hs.191046///Hs.680373///Hs.191046///Hs.680373///Hs.191046///Hs.680373
2924.6	554.2	0.190	3457.0	949.8	0.275	0.232	hydroxysteroid (17-beta) dehydrogenase 2	BC009581	HSD17B2	Cytoplasm	enzyme	NM_002153	Hs.162795///Hs.162795///Hs.162795
1051.2	244.0	0.232	1538.3	343.0	0.223	0.228	ERBB receptor feedback inhibitor 1	BC025337	ERRFI1	Cytoplasm	other	NM_018948	Hs.605445///Hs.605445///Hs.605445
587.8	111.2	0.189	1983.1	454.9	0.229	0.209	guanine nucleotide binding protein (G protein), alpha 14	AF105201	GNA14	Plasma Membrane	enzyme	NM_004297	Hs.657795///Hs.657795///Hs.657795
466.2	95.0	0.204	1193.8	130.1	0.109	0.156	UDP-N-acetyl-alpha-D-galactosamine:polypeptide N-acetylgalactosaminyltransferase-like 2	AL832575	GALNTL2	Cytoplasm	enzyme	NM_054110	Hs.411308///Hs.411308///Hs.411308///Hs.411308
2678.7	323.4	0.121	3166.2	318.1	0.100	0.111	endothelin 1	BC009720	EDN1	Extracellular Space	other	NM_001955	Hs.511899///Hs.700715///Hs.511899///Hs.700715///Hs.511899///Hs.700715

Exp1 and Exp2 indicate the two independent experiments and correspond with Exp1 and Exp2 of Fig. 1b, respectively.

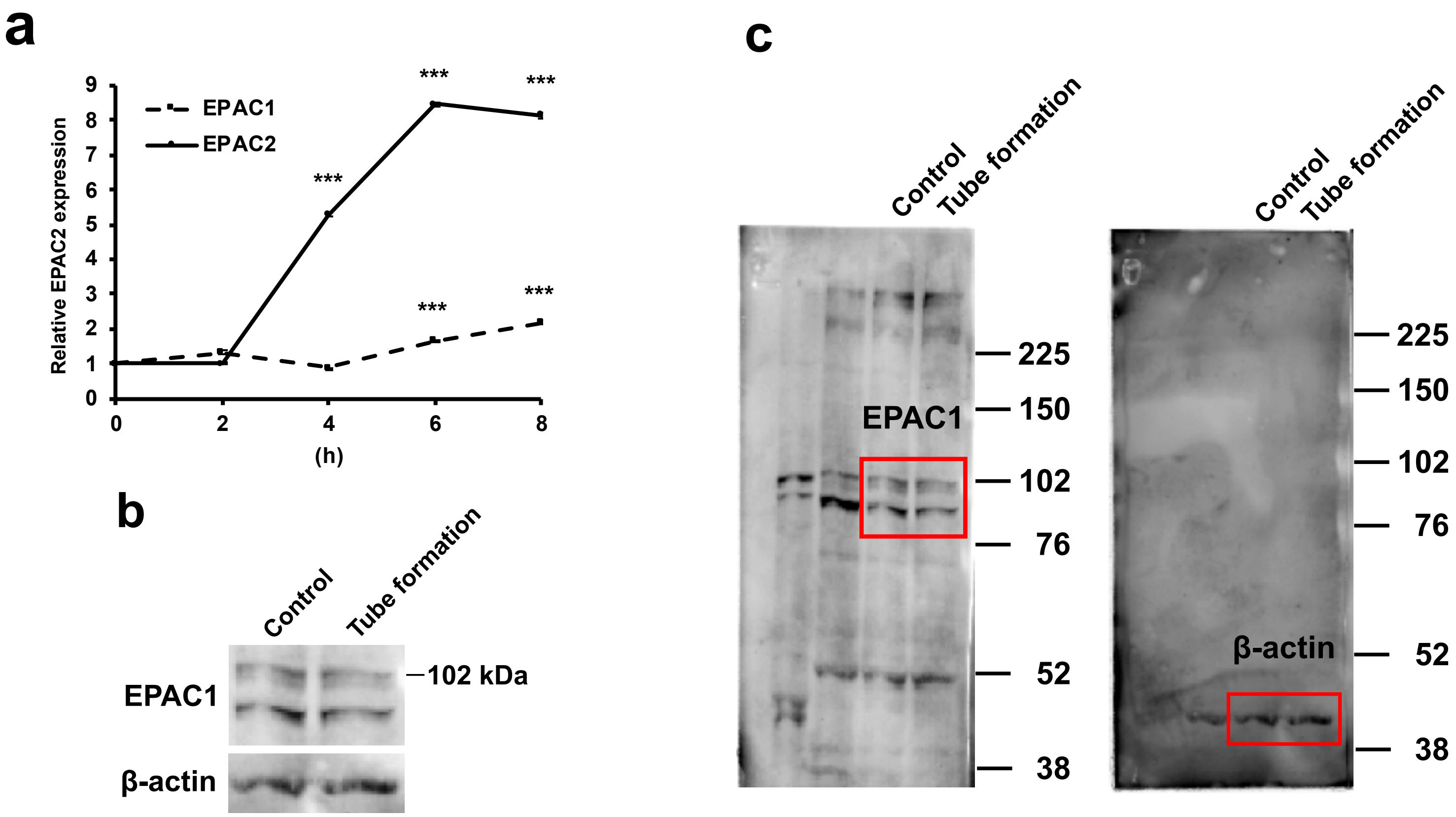

Supplementary Figure S1. (**a**) Time-course experiment of the EPAC1 and EPAC2 expression in endothelial cells during *in vitro* tube formation. HMVECs were cultured on Matrigel in the presence of VEGF, and total RNAs were prepared at the indicated time. EPAC1 mRNA levels were measured using TaqMan Gene Expression Assay (Thermo Fisher Scientific: Hs0018344_m1). Data were expressed as mean ± S.E.M. (n = 3). ***$P < 0.001$ *vs.* 0 h. (**b**) Protein levels of EPAC1 expression. HMVECs were cultured on Matrigel in the presence of VEGF for 8 h, and western blot was performed using antibody specific to EPAC1 (5D3, Cell Signaling Technology). (**c**) Full-length blots of Fig. S1b.

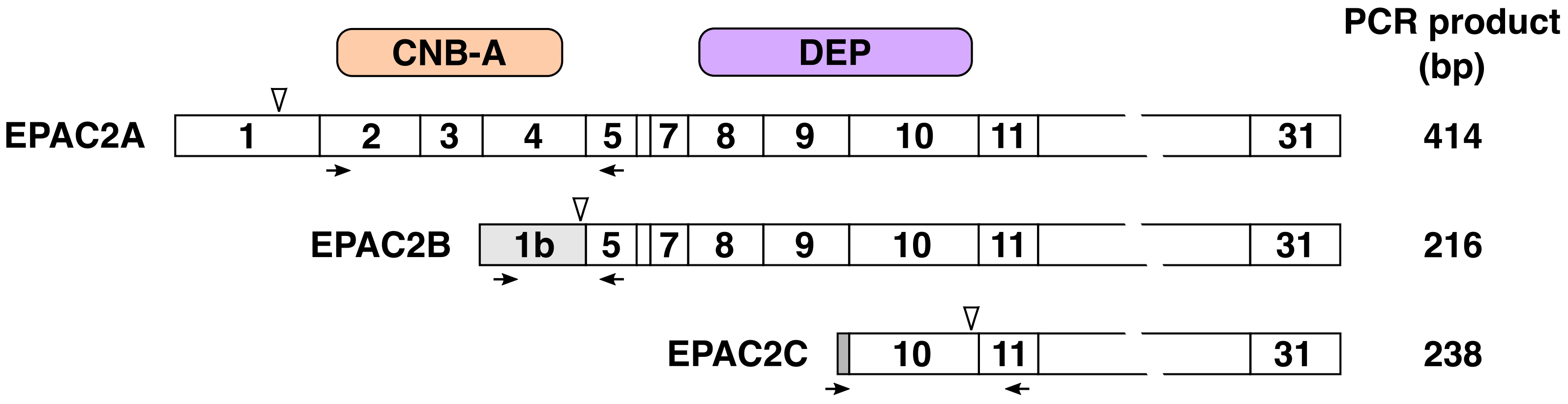

Supplementary Figure S2. The location of primers used for RT-PCR of EPAC2 variants. Exons are numbered and illustrated by white boxes. Introns are illustrated by shaded boxes. The translational start sites (ATGs) are indicated by open arrowheads. The primers for RT-PCR are showed in arrow. EPAC2B has specific exon 1b which is in intron 4 of *RAPGEF4* gene. A forward primer specific to EPAC2B was used together with a reverse primer in exon 5 used for EPAC2A. The specific primers for EPAC2C were positioned in intron 9 and exon 11. Exon 2 to exon 4 of EPAC2A encodes CNB-A domain, and DEP domain is encoded by exon 8 to exon 10 of EPAC2A. EPAC2A has both CNB-A and DEP domains, but EPAC2B lacks CNB-A domain. EPAC2C contains only CNB-B domain in regulatory region.

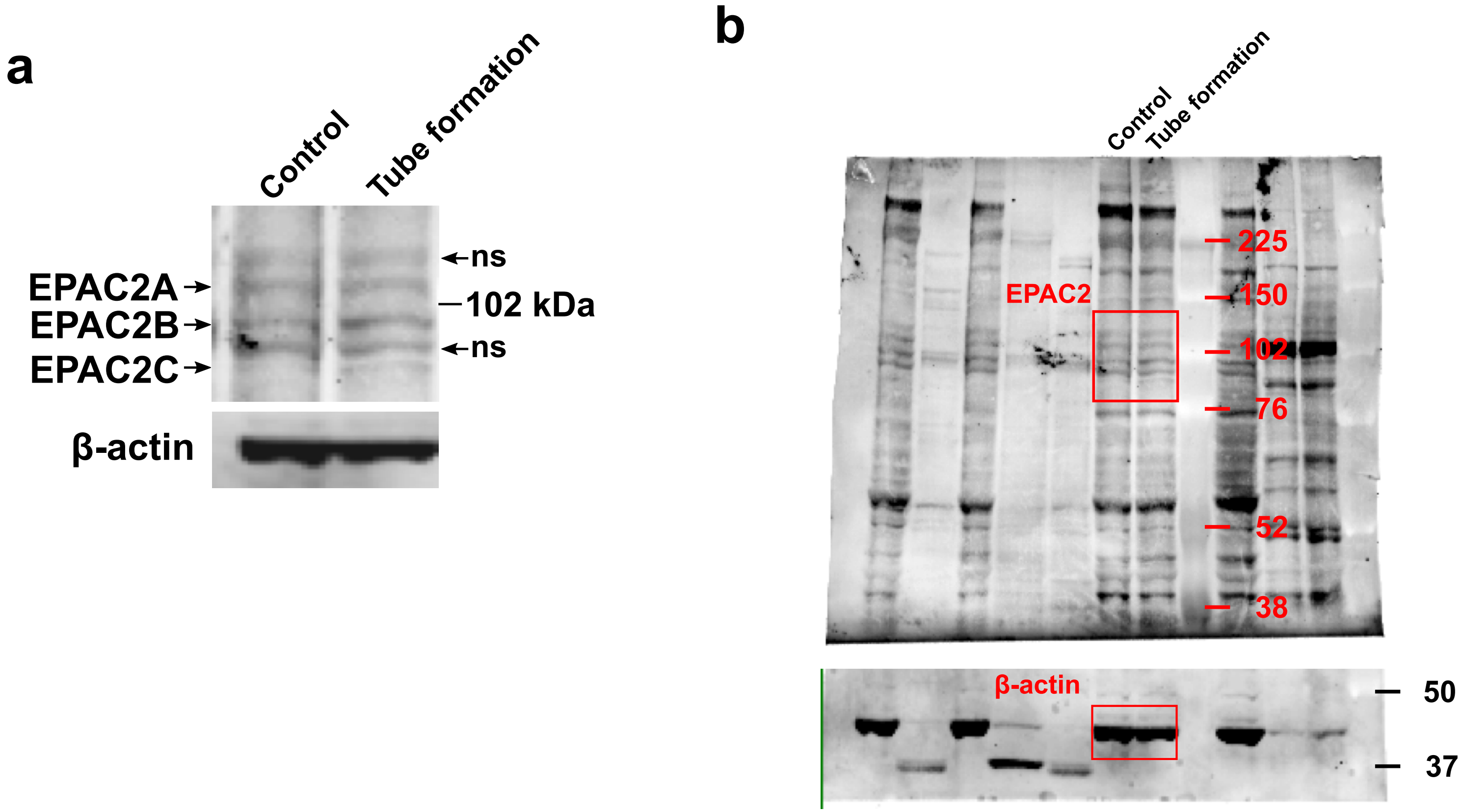

Supplementary Figure S3. Protein levels of EPAC2 expression. (**a**) HMVECs were cultured on Matrigel in the presence of VEGF for 8 h, and western blot was performed using antibody specific to EPAC2 (5B1, Cell Signaling Technology). (**b**) Full-length blots of Fig. S3a.

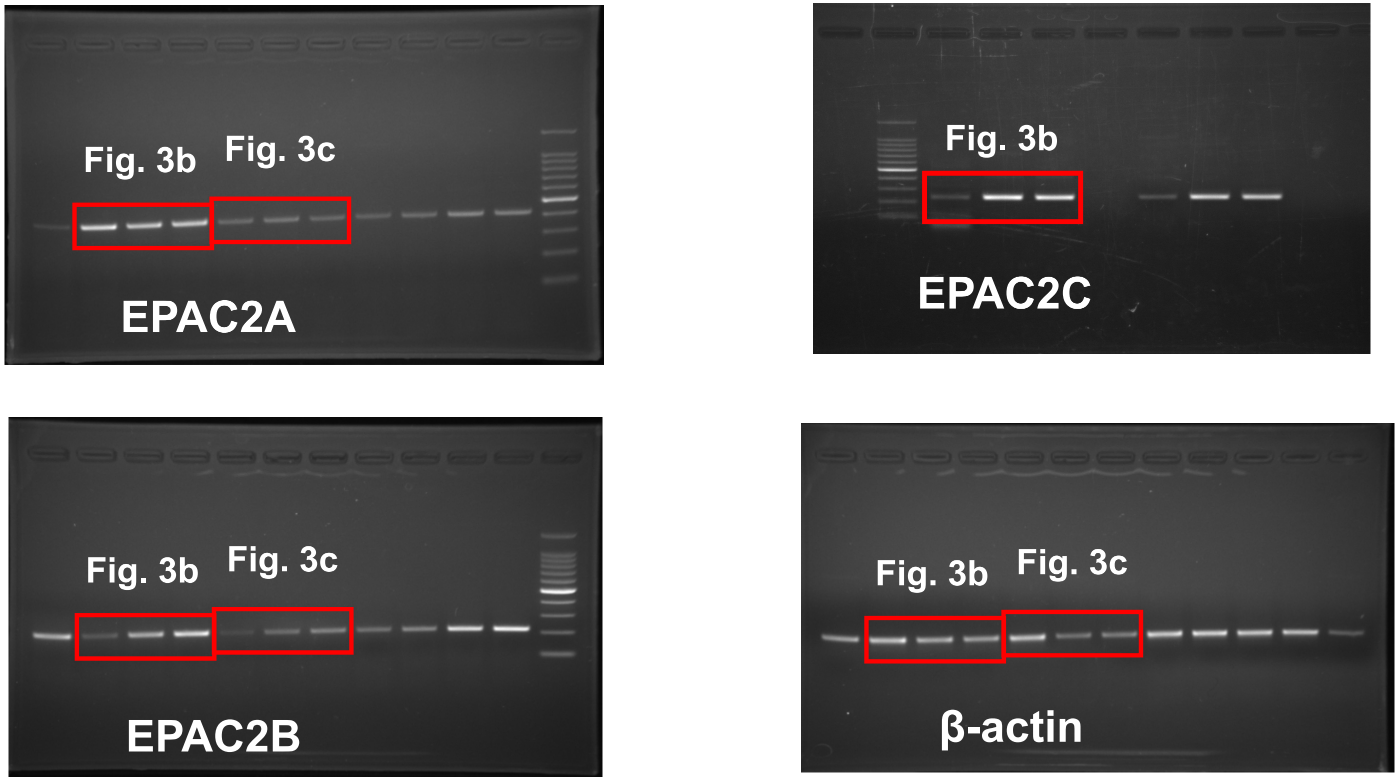

Supplementary Figure S4. Full-length gels of RT-PCR of EPAC2 isoforms of Fig. 3b and 3c. EPAC2A, EPAC2B, and EPAC2C in HMVECs were amplified (Fig. 3b). EPAC2A and EPAC2B in TIME cells were amplified (Fig. 3c).

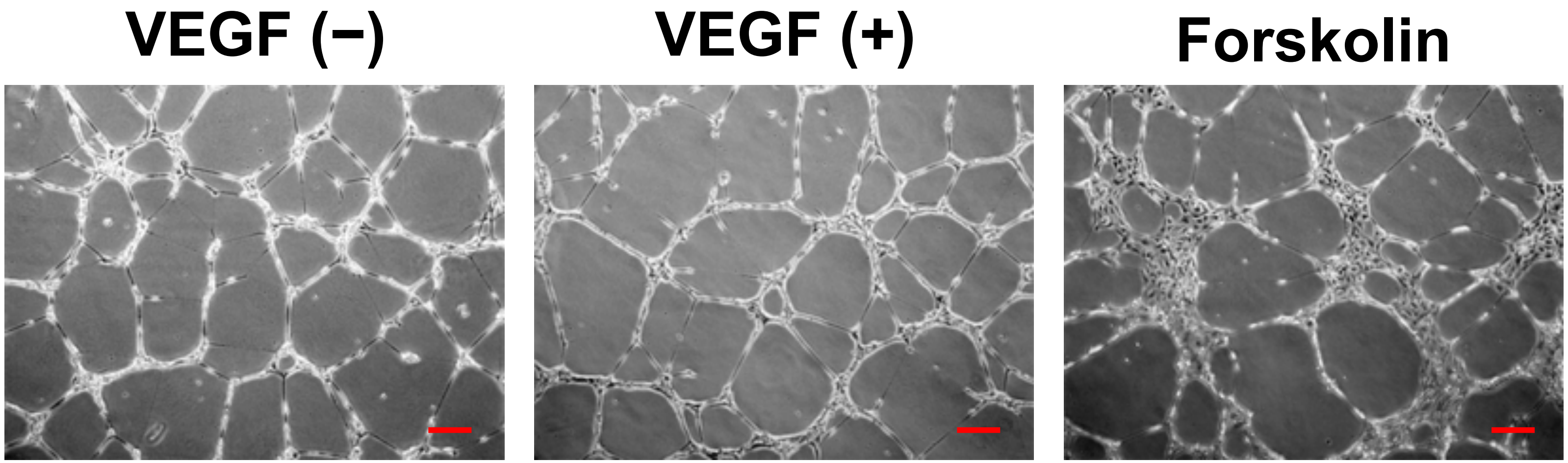

Supplementary Figure S5. HMVECs were cultured on Matrigel containing VEGF (30 ng/mL) or Forskolin (10 μM) for 8 h. Scale bars: 200 μm.

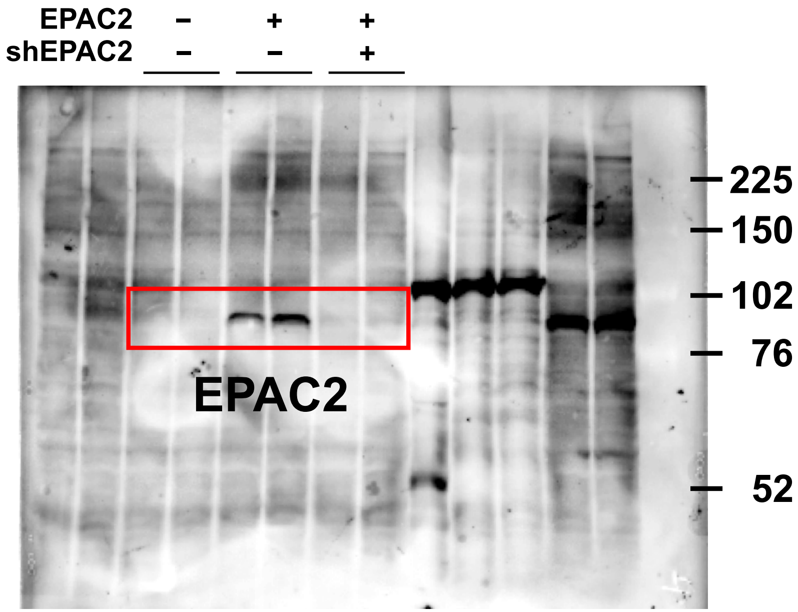

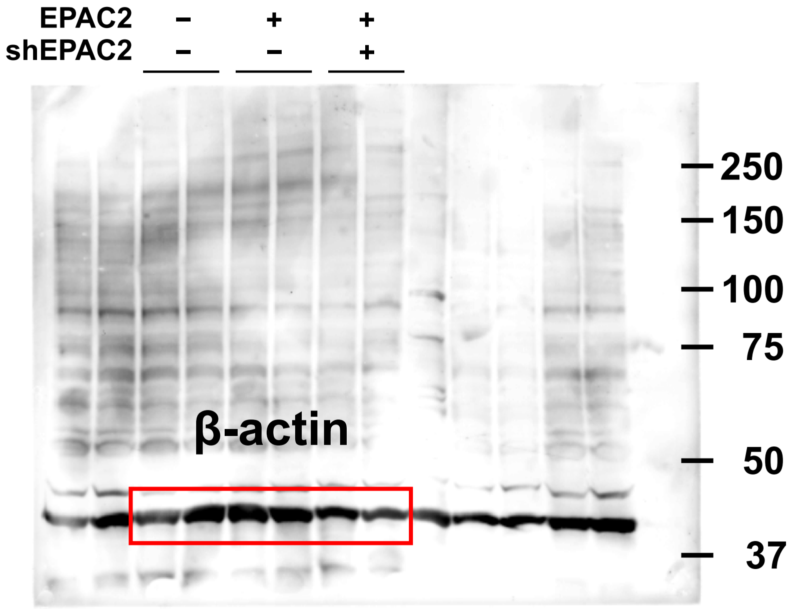

Supplementary Figure S6. Full-length blots of EPAC2 expression of Fig. 4c. EPAC2B was overexpressed in HEK293T cells. Overexpressing EPAC2 in HEK293T cells was knocked down using shEPAC2.
